# Impaired PRC2 activity promotes transcriptional instability and favors breast tumorigenesis

**DOI:** 10.1101/gad.269522.115

**Published:** 2015-12-15

**Authors:** Michel Wassef, Veronica Rodilla, Aurélie Teissandier, Bruno Zeitouni, Nadege Gruel, Benjamin Sadacca, Marie Irondelle, Margaux Charruel, Bertrand Ducos, Audrey Michaud, Matthieu Caron, Elisabetta Marangoni, Philippe Chavrier, Christophe Le Tourneau, Maud Kamal, Eric Pasmant, Michel Vidaud, Nicolas Servant, Fabien Reyal, Dider Meseure, Anne Vincent-Salomon, Silvia Fre, Raphaël Margueron

**Affiliations:** 1 Institut Curie, Paris Sciences et Lettres Research University, 75005 Paris, France;; 2 U934, Institut National de la Santé et de la Recherche Médicale, 75005 Paris, France;; 3 UMR3215, Centre National de la Recherche Scientifique, 75005 Paris, France;; 4 U900, Institut National de la Santé et de la Recherche Médicale, 75005 Paris, France;; 5 Mines ParisTech, 77300 Fontainebleau, France;; 6 Laboratoire de Physique Statistique-Ecole Normale Supérieure de Paris, Centre National de la Recherche Scientifique, 75005 Paris, France;; 7 UMR 8550, Centre National de la Recherche Scientifique, 75005 Paris, France;; 8 Plateforme de PCR Quantitative à Haut Débit Genomic Paris Centre, Institut de Biologie de l’École Normale Supérieure, 75005 Paris, France;; 9 Department of Medical Oncology, Institut Curie, 75005 Paris, France;; 10 EA7285, Université de Versailles, Saint-Quentin-en-Yvelines, 78000 Versailles, France;; 11 UMR_S745, EA7331, Institut National de la Santé et de la Recherche Médicale, 75006 Paris, France;; 12 Facultée des Sciences Pharmaceutiques et Biologiques, Université Paris Descartes, Sorbonne Paris Cité, 75006 Paris, France;; 13 Service de Biochimie et Génétique Moléculaire, Assistance Publique-Hôpitaux de Paris, Hôpital Cochin, 75014 Paris, France;; 14 Platform of Investigative Pathology, 75005 Paris, France

**Keywords:** cancer, chromatin, Polycomb, EZH2

## Abstract

Alterations of chromatin modifiers are frequent in cancer, but their functional consequences often remain unclear. Focusing on the Polycomb protein EZH2 that deposits the H3K27me3 (trimethylation of Lys27 of histone H3) mark, we showed that its high expression in solid tumors is a consequence, not a cause, of tumorigenesis. In mouse and human models, EZH2 is dispensable for prostate cancer development and restrains breast tumorigenesis. High EZH2 expression in tumors results from a tight coupling to proliferation to ensure H3K27me3 homeostasis. However, this process malfunctions in breast cancer. Low EZH2 expression relative to proliferation and mutations in Polycomb genes actually indicate poor prognosis and occur in metastases. We show that while altered EZH2 activity consistently modulates a subset of its target genes, it promotes a wider transcriptional instability. Importantly, transcriptional changes that are consequences of EZH2 loss are predominantly irreversible. Our study provides an unexpected understanding of EZH2's contribution to solid tumors with important therapeutic implications.

## Abstract (precis)

In this study, Wassef et al. used mouse and human models to show that the high expression of Polycomb protein EZH2 in solid tumors is a consequence, not a cause, of tumorigenesis and that low abundance or deletion of EZH2 relative to proliferation is linked to poor prognosis and transcriptional instability.

Eukaryotic cells have developed sophisticated mechanisms to prevent or correct genetic mutations that could result in cell transformation. These mechanisms are often altered during tumor progression, leading to increased genome instability. In addition to genetic lesions, the chromatin undergoes dramatic changes that are routinely used by pathologists to characterize tumor aggressiveness. Consistently, key determinants of chromatin structure and gene regulation are mutated or misregulated in numerous cancer types (You and Jones 2012). Hence, both genetic and epigenetic alterations seem to contribute to deregulation of gene expression programs, favoring the malignant evolution of transformed cells.

The Polycomb group of proteins plays a key role in maintaining transcriptional programs during development (Simon and Kingston 2013), and deregulations of its function has been hypothesized to be involved in cancer (Bracken and Helin 2009). Two multiprotein complexes, Polycomb-repressive complex 1 (PRC1) and PRC2, catalyze a specific modification on the histone tails. The PRC2 complex, through its enzymatic subunits EZH1 and EZH2, is in charge of di- and trimethylation of Lys27 of histone H3 (H3K27me3), a mark linked to transcriptional silencing. Several types of alteration of PRC2 have been reported in tumors. Heterozygous gain-of-function mutations in EZH2 are found in follicular lymphoma and diffuse large cell B-cell lymphoma (Morin et al. 2010), in which the mutant enzyme is proposed to cooperate with its wild-type counterpart to increase the levels of H3K27me3 (Sneeringer et al. 2010). Conversely, loss-of-function mutations in PRC2 genes occur in malignant peripheral nerve sheath tumors (MPNSTs), myelodysplasia, and T-cell acute lymphoblastic leukemia (T-ALL) (Nikoloski et al. 2010; Ntziachristos et al. 2012; De Raedt et al. 2014).

More relevant to the present work, previous studies reported high levels of EZH2 in carcinomas such as prostate and breast cancer (Varambally et al. 2002; Kleer et al. 2003). In these tumor types, high levels of EZH2 are associated with advanced stages of cancer and poor prognosis. Subsequent studies extended these observations to many other tumor types (for review, see Chase and Cross 2011). Overexpression of EZH2 in cancer was proposed to result from gene amplification (Bracken et al. 2003), down-regulation of microRNA 101 (miRNA-101) (Varambally et al. 2008), and stimulation of its expression by the pRB–E2F (Bracken et al. 2003) and MEK–ERK pathways. In addition, the MYC oncogene can also stimulate EZH2 expression (Koh et al. 2011) and has been suggested to interact with the Polycomb machinery at multiple levels in cancer (for review, see Benetatos et al. 2014). Overexpressed EZH2 was proposed to participate in aberrant silencing of tumor suppressor genes such as *DAB2IP* (Min et al. 2010), *ADRB2*, and *SLIT2*.

Paradoxically, recent studies have reported that the levels of H3K27me3 are decreased in several solid tumor types, including breast and prostate (Wei et al. 2008; Holm et al. 2012; Xu et al. 2012; Healey et al. 2014; Bae et al. 2015). Even more surprising, the levels of the enzyme and the mark were found to be anti-correlated between the different breast cancer subtypes (Holm et al. 2012), and, while high expression of EZH2 correlates with poor prognosis, high levels of H3K27me3 correlate with good prognosis (Holm et al. 2012; Bae et al. 2015). This has led several groups to propose that EZH2 might play PRC2-independent roles in carcinomas (Lee et al. 2011; Xu et al. 2012). However, no clear picture has emerged from these studies on the involvement of EZH2 in solid tumors. Thus, whether elevated expression of EZH2 in carcinomas actively contributes to tumor progression or is simply a consequence of malignant evolution remains an open question.

Here, we set out to investigate the role of EZH2 in carcinomas using genetic tools in mouse and human model systems. We discovered that Ezh2 is largely dispensable for development of solid tumors and that the absence of the enzyme can actually enhance tumorigenesis. Consistently, when corrected for proliferation, the prognostic value of EZH2 expression is inverted; low EZH2 expression relative to proliferation is associated with poor prognosis in breast cancer. In addition, we found that mutations in PRC2 genes are linked to poor prognosis and are found in breast cancer metastases. Importantly, we showed that impaired PRC2 activity promotes transcriptomic instability with irreversible consequences on the gene expression program. Altogether, our study sheds a new light on the interplay between the Polycomb machinery and cancer and calls for caution concerning disruption of PRC2 as a therapeutic strategy.

## Results

### Ezh2 is dispensable in genetically engineered mouse models of prostate and breast cancers

Given the prior links made between Ezh2 overexpression and the more aggressive forms of prostate cancer (Varambally et al. 2002, 2008), we used genetically engineered mouse models of prostate cancer to investigate the role of the enzyme in carcinogenesis. Both amplification of the *c-MYC* oncogene and loss of the *PTEN* tumor suppressor are common features of human prostate cancer, and corresponding alterations in the mouse prostate result in adenocarcinomas.

**Figure 1.**
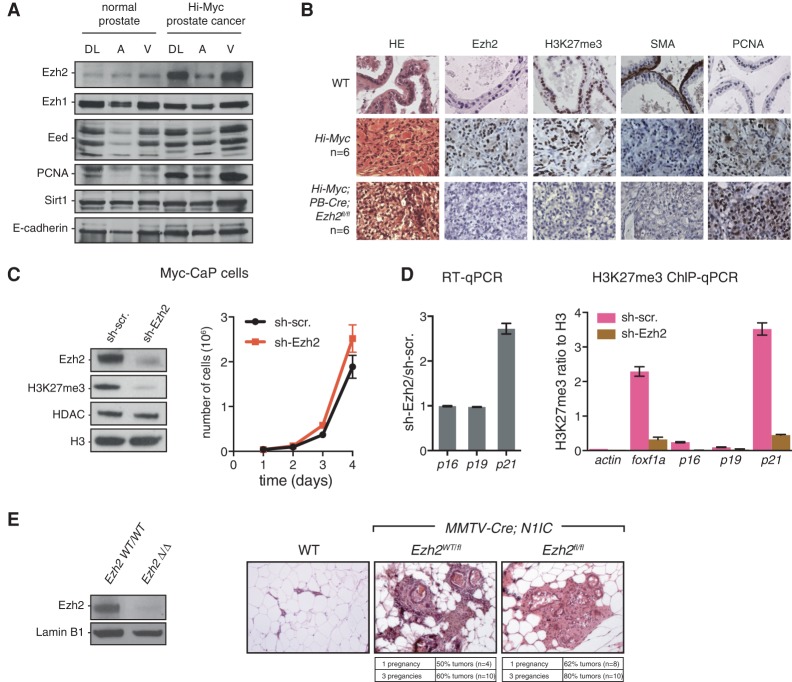
Ezh2 is dispensable in genetically engineered mouse models of prostate and breast cancers. (*A*) Western blot on whole-tissue lysates from dorso–lateral (DL), anterior (A), and ventral (V) lobes of a normal prostate and a Hi-Myc invasive prostate cancer. The specific antibodies used are indicated at the *left*. (*B*) Hematoxylin and eosin (HE) and IHC staining of different proteins, as indicated, in a normal mouse prostate (wild type) and *Hi-Myc* and *Hi-Myc;PB-Cre;Ezh2*^*fl/fl*^ invasive prostate cancer. (*C*) Impact of Ezh2 knockdown in Myc-CaP cells (derived from Hi-Myc mouse prostates) on Ezh2, H3K27me3, HDAC, and H3 (Western blot; *left* panel) as well as cell proliferation (*right* panel). Mean ± SD. *n* = 3. (*D*, *left* panel) RT-qPCR analysis of *p16*, *p19*, and *p21* expression in Myc-CaP cells. RT-qPCR values indicate relative expression in sh-Ezh2 compared with sh-scramble cells after normalization to TBP. (*Right* panel) Enrichment of H3K27me3 by chromatin immunoprecipitation (ChIP) and qPCR (ChIP-qPCR) at the corresponding loci. *actin* and *foxf1a* were used as negative and positive controls, respectively. ChIP-qPCR values indicate relative enrichment compared with histone H3. Mean ± SD. *n* = 3. (*E*, *left* panel) Western blot showing loss of Ezh2 protein in *MMTV-Cre;N1IC;Ezh2*^*fl/fl*^ FACS-sorted luminal cells. *MMTV-Cre;N1IC;Ezh2*^*wt/wt*^ cells were used as a control. (*Right* panel) Representative HE staining on mammary glands of wild-type, *MMTV-Cre;N1IC;Ezh2*^*wt/fl*^, and *MMTV-Cre;N1IC;Ezh2*^*fl/fl*^ mice showing the presence of tumors in the presence or absence of Ezh2.

We first examined 9- to 12-mo-old Hi-Myc mice, driving c-Myc expression in the prostate, which developed invasive prostate adenocarcinomas with 100% penetrance (*n* = 6). These mice exhibited high levels of Ezh2 and proliferation marker PCNA relative to normal prostates, as shown by both Western blot and immunohistochemistry (IHC) (Fig. 1A,B). Unlike Ezh2, the expression of Ezh1 did not significantly change, while Eed and Suz12, two core PRC2 components, were modestly up-regulated (Fig. 1A; Supplemental Fig. S1A). This Hi-Myc mouse line was then crossed to an *Ezh2* conditional knockout mouse (Su et al. 2003), and genetic deletion of *Ezh2* was induced in prostate epithelium with a *Probasin*-driven Cre recombinase (PB4-Cre) (Wu et al. 2001). Of note, presumably due to the postnatal expression of the Cre, prostate-specific deletion of *Ezh2* in *PB4-Cre;Ezh2*^*fl/fl*^ males had no noticeable consequences on normal prostate tissue (data not shown). Ezh2 was efficiently depleted in *Hi-Myc;PB4-Cre;Ezh2*^*fl/fl*^, as assessed by IHC (Fig. 1B) and Western blot (Supplemental Fig. S1B). Importantly, although H3K27me3 was heavily reduced in tumors lacking Ezh2 in 9- to 12-mo-old mice (Fig. 1B), invasive adenocarcinomas still formed with full penetrance (*n* = 6). The invasiveness is evidenced by the disruption of the fibromuscular layer stained by smooth muscle actin (SMA) (Fig. 1B). In addition, *Ezh2* knockout tumors, like *Ezh2* wild-type tumors, retained high levels of PCNA (Fig. 1B), androgen receptor (AR) (Supplemental Fig. S1C), and the epithelial marker E-cadherin (Supplemental Fig. S1C) and were negative for the expression of the tumor suppressor Nkx3.1 (Supplemental Fig. S1C), as previously shown for Hi-Myc tumors (Ellwood-Yen et al. 2003). To determine whether tumors progressively adapt to lack of Ezh2 or whether Ezh2 is overall dispensable in this model, we knocked down Ezh2 through shRNA interference in a cell line derived from advanced Hi-Myc tumors (Myc-CaP) (Watson et al. 2005). Despite strong down-regulation of H3K27me3, proliferation was unimpaired. Prior studies suggest that Ezh2 can control cell proliferation in part through silencing of the *Ink4a/Arf* and *p21* tumor suppressor loci (Bracken et al. 2007; Seward et al. 2013). However, the levels of *p16/p19* transcripts, already detectable in sh-scramble Myc-CaP cells, were not affected upon Ezh2 knockdown in this model (Fig. 1C,D; data not shown). The *p21* transcript was nevertheless significantly up-regulated (Fig. 1D). H3K27me3 was present at low, close to background, levels at the *Ink4a/Arf* locus in comparison with *foxf1a* (an established PRC2 target) and *p21* loci (Fig. 1D, right panel). Thus, in the context of c-Myc-induced prostate cancer, cell proliferation and malignant evolution appear unaffected by the absence of Ezh2.

We analyzed a second model of prostate cancer, generated by deletion of the *Pten* tumor suppressor. Conditional deletion of this gene in mouse prostates leads to prostate adenocarcinomas with varying degrees of severity (Wang et al. 2003; Ma et al. 2005), presumably due to differences in the genetic background and/or mutant allele used. In our mixed strain, PB4-Cre-induced deletion of *Pten* led to intraepithelial neoplasia at 6–9 mo of age showing no sign of invasion (*n* = 7) (Supplemental Fig. S1D). Relative to normal prostates, Ezh2 expression was nonetheless up-regulated in these tumors (Supplemental Fig. S1D, left panel). However, similar to the Hi-Myc model, deletion of *Ezh2* did not prevent tumor development (*n* = 7) (Supplemental Fig. S1D, right panel).

Since high EZH2 expression has also been reported in breast cancer (Kleer et al. 2003), we next turned to a mouse model eliciting mammary tumors upon mammary-specific expression of the activated form of Notch1 (N1ICD). Aberrant Notch signal activation is a common feature of human breast cancers (Stylianou et al. 2006) and has been shown to induce mammary tumors in mice (Bolos et al. 2013). We induced ectopic Notch activation by targeting an inducible *Rosa26*^*flox*^*N1ICD*^*flox*^ allele (Murtaugh et al. 2003) to the mammary epithelium with MMTV-Cre mice, as previously described (Bolos et al. 2013). *Rosa26N1ICD;MMTV*-*Cre* compound female mice developed hormone-dependent mammary tumors. They were subjected to one or three rounds of pregnancy and analyzed for the presence of tumors. In this model, penetrance of tumor development was incomplete even after three rounds of pregnancy. Mammary-specific deletion of *Ezh2* did not impair tumor development but in fact resulted in an increased penetrance of tumor formation (Fig. 1E).

Altogether, our findings based on three different mouse models indicate that solid tumors can develop in the absence of Ezh2.

### H3K27me3 homeostasis is compromised in breast cancer

Since Ezh2 is dispensable for mouse prostate and mammary cancer development, we wondered why the enzyme is nonetheless highly up-regulated in tumors. High EZH2 expression has been repeatedly found to be associated with proliferating tissues (e.g., Margueron et al. 2008), and its expression was shown to be under the influence of key cell proliferation pathways (Bracken et al. 2003). In addition, EZH2 expression in several solid tumor types was shown to be correlated with proliferation (Bachmann et al. 2006). Thus, elevated expression of EZH2 in cancer may simply result from abnormally high cell proliferation rates in tumors rather than deregulated expression.

**Figure 2.**
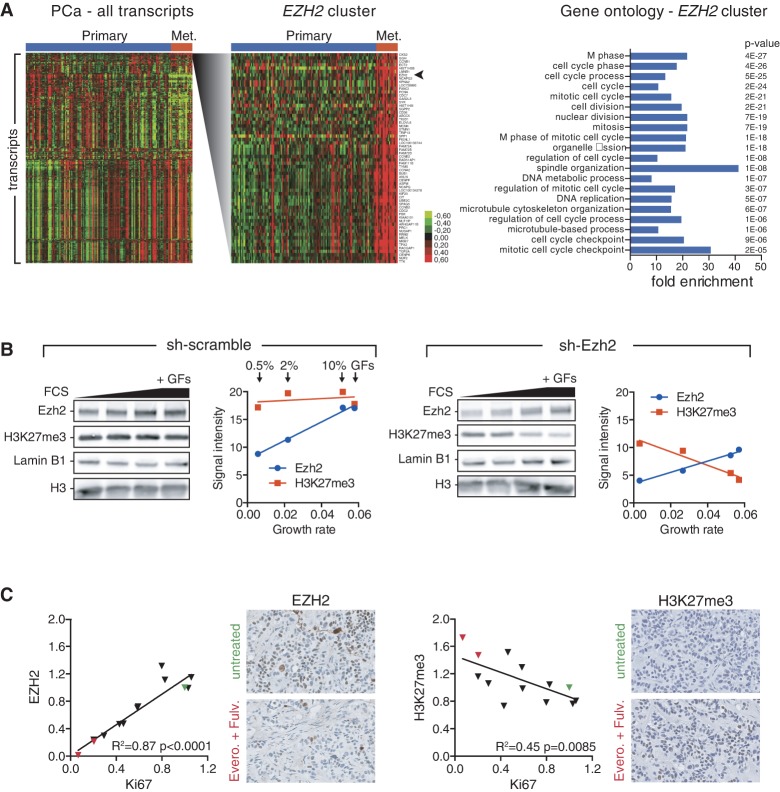
Coupling of EZH2 expression to proliferation is required for H3K27me3 homeostasis but perturbed in breast cancers. (*A*, *left* panel) Heat map of hierarchical clustering of the most significantly varying transcripts in primary and metastatic (Met.) prostate cancers (PCa). Data are from Taylor et al. (2010). Samples are arranged horizontally, and transcripts are arranged vertically. The cluster containing the *EZH2* transcript is shown in greater detail at the *right*. (*Right* panel) Gene ontology (DAVID, http://david.abcc.ncifcrf.gov) of the *EZH2* cluster showing the 20 most significantly enriched categories, their fold enrichment, and corresponding *P*-values. (*B*) Western blot probed with antibodies recognizing Ezh2, H3K27me3, Lamin B1, or histone H3 in sh-scramble (*left* panel) and sh-Ezh2 (*right* panel) Myc-CaP cells. In order to modulate proliferation in vitro, cells were cultured in the presence of 0.5%, 2%, or 10% fetal calf serum (FCS) or 10% FCS medium plus a cocktail of growth factors (bovine pituitary extract, insulin, and epidermal growth factor, indicated as +GFs in the last lane). Corresponding dot plots show signal quantification of Ezh2 (blue) and H3K27me3 (red) abundance (arbitrary units) as a function of the growth rate (number of divisions per cell per hour), as assessed by proliferation assays carried out in parallel for each culture condition. (*C*) EZH2 and H3K27me3 IHC staining quantifications across two patient-derived xenografts (PDXs) treated with various combinations of drugs. Correlation plots of EZH2 versus Ki67 and H3K27me3 versus Ki67 signal intensities are shown. Intensity values were normalized to the control (untreated) condition (green triangles). The everolimus + fulvestrant-treated PDXs show strongly reduced proliferation (red triangles). Each dot corresponds to the mean of six measurements (two stainings on three biological replicates). The corresponding coefficient of determination (*R*^2^) and *P*-value of the linear regression are shown. Representative IHC staining for EZH2 and H3K27me3 in untreated and everolimus + fulvestrant treated PDXs are shown. Nuclei are counterstained in blue/purple.

To obtain further insight into the expression of EZH2 in cancer, we analyzed transcriptome data from a publicly available study on 131 primary prostate tumors and 19 metastases (Taylor et al. 2010). As expected, hierarchical clustering of the transcriptome data revealed that the *EZH2* transcript is part of a cluster of genes highly expressed in metastatic prostate cancer (Fig. 2A). Importantly, cell cycle and proliferation genes (e.g., *Ki67* and *PCNA*) are overly represented in this cluster. It is noteworthy that several transcripts (e.g., *Ki67*) (Supplemental Fig. S2A) display stronger differential expression between primary and metastatic cancer than *EZH2*. This further suggests that association of high *EZH2* with cancer aggressiveness might reflect the increased cell proliferation occurring in advanced stages of prostate cancer (e.g., see Tomlins et al. 2007). A similar analysis on a breast cancer cohort comprising 146 samples from the four main molecular subtypes (Maire et al. 2013) confirmed that EZH2 expression correlates with proliferation markers (Supplemental Fig. S2B). In addition, analysis of copy number data from the same cohort revealed that amplification of *EZH2* is a rare event, since no instances were found in this data set. Gains occur in proportions similar to losses (Supplemental Fig. S2C; data not shown), arguing against a major role for copy number gains or amplifications in driving high EZH2 levels. Thus, high EZH2 expression seems to be predominantly linked to proliferation.

To assess why EZH2 expression is associated with cell proliferation, we turned to a cell-based system allowing modulation of proliferation rate through increased serum concentration and addition of growth factors. Modulation of Myc-CaP proliferation in vitro revealed that, while Ezh2 expression shows a near-perfect correlation to the rate of cell division, H3K27me3 remains constant (Fig. 2B, left). This result is consistent with a previous study monitoring EZH2 and H3K27me3 upon serum stimulation of quiescent cells (Hansen et al. 2008). It further suggests that proliferation-induced Ezh2 levels may serve to oppose cell division-mediated dilution of H3K27me3. To test this hypothesis, we altered Ezh2 expression using shRNA-mediated knockdown. Ezh2 expression was reduced as expected, but, more importantly, the rate of increase of Ezh2 with proliferation was also diminished (Supplemental Fig. S2D). This resulted in a gradual drop of H3K27me3 (Fig. 2B, right), suggesting that the increase of Ezh2 was no longer sufficient to counteract cell division-mediated dilution of the mark. Thus, while Ezh2 is not an obligate modulator of cell proliferation, the tight coupling of Ezh2 expression levels to the rate of cell division is required to ensure homeostatic maintenance of H3K27me3.

This result prompted us to hypothesize that the anti-correlated levels of EZH2 and H3K27me3 observed in several solid tumor types might stem from a failure to properly counteract cell division-mediated dilution of the histone mark. We thus sought to assess the impact of modulating proliferation on the levels of EZH2 and H3K27me3 in the context of human breast cancers. We analyzed EZH2 and H3K27me3 levels by IHC in two previously characterized patient-derived xenografts (PDXs) of estrogen-positive breast cancer (Cottu et al. 2014). The engrafted mice were treated with various combinations of endocrine therapies and the mTOR inhibitor everolimus, the impact of which on tumor proliferation was evaluated by Ki67 staining (Cottu et al. 2014). As previously reported (Supplemental Table S1; Cottu et al. 2014), some drug combinations led to a near complete inhibition of cell proliferation (e.g., everolimus + fulvestrant), while other treatments only reduced proliferation (e.g., everolimus alone or everolimus + tamoxifen) or failed to impair proliferation (e.g., ovariectomy). Quantification of EZH2 signal revealed that it was highly correlated to Ki67 (Fig. 2C, left; Supplemental Table S1), confirming that, in the context of tumors, EZH2 expression is under the control of proliferation cues. Importantly, though, H3K27me3 signal was significantly anti-correlated to both Ki67 (Fig. 2C, right) and EZH2 (Supplemental Fig. S2E).

Although the drugs used are likely to impact processes other than proliferation, which might lead to confounding effects on H3K27me3 homeostasis, these data suggest that, in spite of higher EZH2 levels, the PRC2 complex might not be able to match the abnormally high proliferation of breast cancer cells, leading to down-regulation of H3K27me3.

### Genetic loss of *EZH2* is linked to poor prognosis in breast cancer

Our results question the contribution of proliferation to EZH2's prognostic value. Indeed, such an association was found in many gene expression-based signatures associated with clinical outcome (Venet et al. 2011).

**Figure 3.**
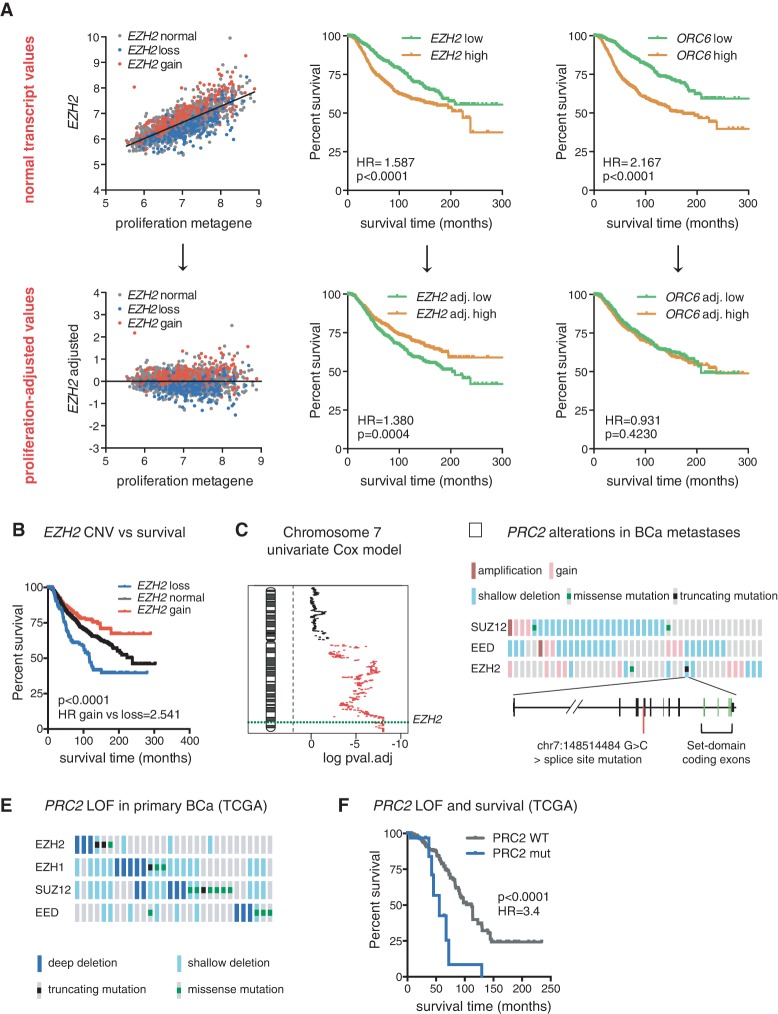
Genetic loss of *EZH2* is linked to poor prognosis in breast cancer. (*A*, *top left* panel) Correlation plot of *EZH2* transcript and a proliferation metagene. Residual (adjusted) values of *EZH2* transcripts to the proliferation metagene are shown in the *bottom left* panel. *EZH2* copy number variations are color-coded, with normal copy number in gray, hemizygous loss in blue, and gain in red. The same procedure was applied to adjust *ORC6* transcript values (data not shown). Kaplan-Meier plots of breast cancer-specific survival for patients with primary tumors with high (above median) or low (below median) *EZH2* and *ORC6* transcript levels are shown in the *middle* and *right top* panels. Kaplan-Meier plots of breast cancer-specific survival for patients with primary tumors with high versus low proliferation-adjusted levels of *EZH2* and *ORC6* transcripts are shown in the *middle* and *right bottom* panels. The hazard ratio (HR) between the highest and lowest survival groups and *P*-values are displayed on Kaplan-Meier plots. (*B*) Kaplan-Meier plot of breast cancer-specific survival for patients with primary tumors with normal *EZH2* or hemizygous loss or gain of *EZH2*. (*C*) Univariate analysis showing the association between genetic loss and death from breast cancer on all genes of chromosome 7. False discovery rate (FDR)-corrected *P*-values (log_10_ scale) are plotted for all chromosome 7 genes, and significant values are highlighted in red (threshold of 0.15). A dashed green line indicates the position of the *EZH2* locus. The analyses shown in *A*–*C* were performed on data from 2000 primary breast cancers of the METABRIC cohort. (*D*, *top*) Oncoprint generated on the cBioPortal OncoPrinter showing genomic alterations and mutations in genes encoding PRC2 core components in 58 breast cancer (BCa) metastases. Only altered cases are shown. (*Bottom*) Schematic representation of the *EZH2* locus showing the position of a splice site mutation in position −1 of exon 11. (*E*) Oncoprint (cBioPortal) showing loss-of-function (LOF) mutations of core PRC2 genes in The Cancer Genome Atlas (TCGA) breast cancer data set. (*F*) Kaplan-Meier plot of overall survival associated with the corresponding tumors compared with the remaining (PRC2 wild-type) tumors.

To address this issue, we used transcriptome/CNV data from the Molecular Taxonomy of Breast Cancer International Consortium (METABRIC) (Curtis et al. 2012), which collected information on transcript levels and copy number as well as long-term clinical follow-ups from 2000 breast cancers (Curtis et al. 2012). We first investigated the prognostic value of the *EZH2* transcript in comparison with that of the *ORC6* transcript (a proliferation-associated transcript used as a control) and a proliferation metagene consisting of the median expression of 54 proliferation-associated transcripts used as a molecular readout for proliferation (Nagalla et al. 2013). As expected, all three variables displayed a significant association with outcome as assessed by Receiver Operating Characteristic (ROC) analysis (Supplemental Fig. S3A) or Kaplan-Meier analysis (Fig. 3A, top panels), although the prognostic value of *EZH2* expression was the least powerful. We then sought to evaluate the prognostic value of the *EZH2* and *ORC6* transcripts independently of proliferation. For this purpose, we calculated residual (“adjusted”) values of *EZH2* and *ORC6* to the proliferation metagene (shown for *EZH2* in Fig. 3A, left panels). Strikingly, adjusted *EZH2* was now negatively associated with outcome (Fig. 3A, bottom middle panel; Supplemental Fig. S3B), suggesting that low *EZH2* expression relative to proliferation is linked to a poor prognosis. By comparison, the adjusted *ORC6* transcript no longer bore any association with outcome (Fig. 3A, bottom right panel; Supplemental Fig. S3C), confirming that its prognostic value is mainly proliferation-dependent. Thus, the association of *EZH2* expression with prognosis comprises both a positive component linked to proliferation and a negative component independent of proliferation.

Copy number variations seemed to largely account for variations of *EZH2* levels independently of proliferation (Fig. 3A). We therefore stratified tumors according to the copy number status of the gene. Hemizygous loss of *EZH2* was indeed linked to a significantly worse prognosis in comparison with normal *EZH2* copy number (Fig. 3B). Conversely, gain of *EZH2* was associated with better prognosis than normal *EZH2* copy number*.* Association of *EZH2* CNV with outcome was independent of estrogen status (Supplemental Fig. S3D,E), although more pronounced in estrogen-positive tumors. In order to determine the size of the region surrounding *EZH2* showing a correlation between genetic loss and outcome, we analyzed the prognostic association of all annotated genes on chromosome 7. Loss of the long arm of chromosome 7 was significantly linked to poor prognosis, with the end of the arm (encompassing the *EZH2* locus) having the strongest association (Fig. 3C).

We next assessed alterations of *EZH2* or other genes encoding core PRC2 components by targeted sequencing in breast cancer metastases previously analyzed by Affymetrix CytoScan arrays (Le Tourneau et al. 2014). Interestingly, in addition to metastases having missense mutations in *SUZ12* (two samples) and *EZH2* (one sample), one metastasis harbored a critical splice site mutation in *EZH2* in the −1 position relative to exon 11 (Fig. 3D; Supplemental Table S2). This mutation is predicted to abolish splicing at this intron/exon junction and to result in a truncated protein lacking the catalytic SET domain. Since it is found in a metastasis having a hemizygous loss of *EZH2*, this mutation could drive a complete PRC2 loss of function.

Finally, we investigated the presence of homozygous loss or mutation of PRC2 core component genes in The Cancer Genome Atlas (TCGA) breast cancer cohort. Strikingly, the group of tumors harboring such mutations (3% of all tumors) (Fig. 3E) displayed a significantly worse prognosis than the non-PRC2 mutant tumors (Fig. 3F).

In summary, our data show that the association of EZH2 expression to prognosis results from its correlation to proliferation. Strikingly, low levels of EZH2 relative to proliferation, resulting from genetic loss of the gene and mutations in PRC2 genes, are in fact associated with poor prognosis.

### Genetic disruption of *EZH2* in a breast cancer cell line promotes tumorigenesis

**Figure 4.**
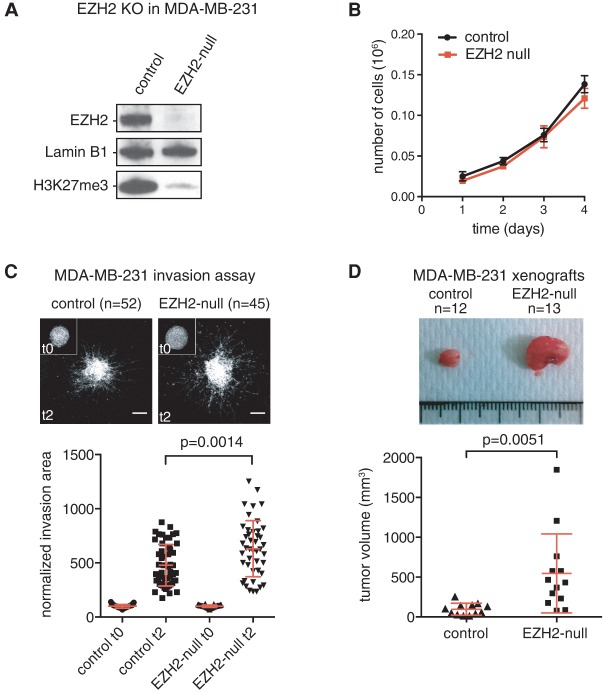
Genetic disruption of *EZH2* in a breast cancer cell line promotes tumorigenesis. (*A*) Western blot showing loss of the EZH2 protein and H3K27me3 mark in the EZH2 full knockout MDA-MB-231 cell line (indicated as EZH2-null) compared with the parental clone mutant for one allele out of three. (*B*) Proliferation curve of control and EZH2-null cells. (*C*) Multicellular spheroids of control or EZH2-null MDA-MB-231 cells were embedded in 3D acid-extracted type I collagen (T0) and further incubated for 2 d (T2). Images show representative phalloidin-labeled spheroids collected at T0 (*inset*) or T2. Bars, 200 μm. Data represent mean invasion area in type I collagen at T2 normalized to the mean invasion area at T0 ±SEM. *n* = 3; 15–20 spheroids were analyzed for each cell line, with a total of 52 and 45 measurements for control and EZH2-null cells, respectively. Red bars indicate mean ± SD. The *P*-value of the two-tailed unpaired *t*-test is indicated. (*D*) Representative pictures of orthotopic tumor xenografts developed from the control MDA-MB-231 clone and EZH2-null clone (*top* panel) and a plot showing corresponding tumor volumes (*bottom* panel). Red bars indicate mean ± SD. The *P*-value of the two-tailed unpaired *t*-test is indicated.

Since our analyses indicated that impaired PRC2 function is associated with poor prognosis, we used CRISPR/CAS9-based genome engineering tools to delete *EZH2* in a cellular model of human breast cancer. We chose the MDA-MB-231 cell line, a widely studied near-triploid cell line derived from a metastatic triple-negative (estrogen-, progesterone-, and HER2-negative) breast cancer. We sequentially targeted all three alleles of *EZH2* and confirmed that the resulting cell line no longer expressed EZH2 when compared with the parental clone carrying only one mutant allele, leading to a near-complete erasure of H3K27me3 (Fig. 4A). In contrast to a previous report using RNAi (Gonzalez et al. 2009), loss of EZH2 did not have an impact on cell proliferation (Fig. 4B). However, we observed an increased three-dimensional (3D) cell migration through type I collagen, indicative of metastatic potential (*n* = 52 for control cells, and *n* = 45 for *EZH2*-null cells) (Fig. 4C). Importantly, we confirmed these results on proliferation and 3D cell migration using an inhibitor targeting both EZH1 and EZH2 (Supplemental Fig. S4; Konze et al. 2013), indicating that genetic deletion of EZH2 recapitulates pharmacological inhibition of the enzyme and that EZH1 does not compensate for loss of EZH2 in this model. Finally, we analyzed the consequences of *EZH2* deletion on orthotropic tumor growth in mammary fat pads of immune-deficient host mice. Strikingly, tumors originating from *EZH2*-null xenografts were significantly bigger than the control tumors (*n* = 12 for control xenografts, and *n* = 13 for *EZH2*-null xenografts) (Fig. 4D).

These results suggest that PRC2-mediated gene silencing might have a protective function in breast tumorigenesis.

### Impaired PRC2 function selectively affects H3K27me3-low genes

**Figure 5.**
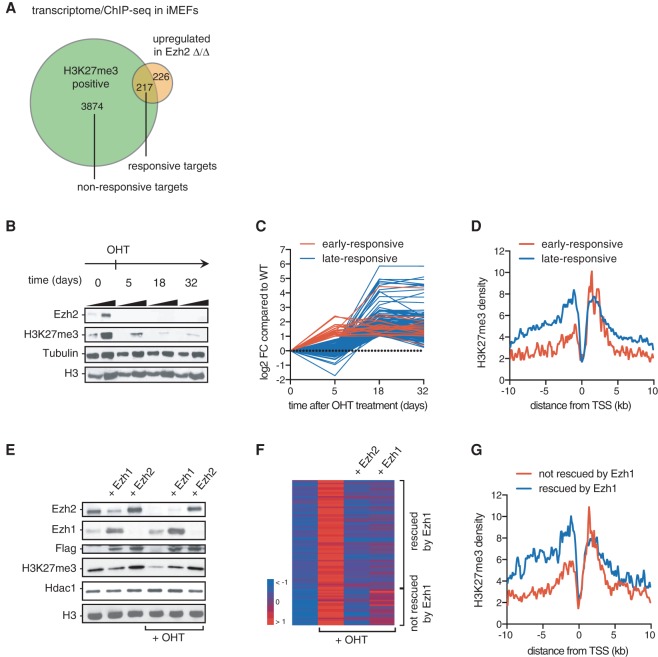
Impaired PRC2 function selectively affects H3K27me3-low genes. (*A*) Venn diagram showing the overlap between up-regulated transcripts upon *Ezh2* deletion and Ezh2 targets defined by the presence of H3K27me3 in the promoter of wild-type versus Ezh2^Δ/Δ^ iMEFs. Up-regulated transcripts were identified with a minimum adjusted *P*-value of 15% and a minimum fold change of two. Targets that are H3K27me3-enriched and up-regulated upon loss of Ezh2 (*n* = 217) are defined as “responsive targets” as opposed to H3K27me3-enriched “nonresponsive targets” (*n* = 3974). Genes that are up-regulated but are H3K27me3-negative (*n* = 226) correspond to indirect targets. (*B*) Time-course analysis by Western blot of Ezh2 and H3K27me3 before (time 0) and at different times after OHT-induced *Ezh2* deletion. A two-point titration is provided for each condition. Tubulin and H3 were used as loading controls. (*C*) Time-course expression analysis of Ezh2-responsive direct targets. Red represents early responsive targets, and blue indicates late responsive genes. All values were normalized to initial (time 0) values. (*D*) H3K27me3 density plot around the transcription start site (TSS) of early responsive (in red) and late responsive targets (in blue). (*E*) Western blot of control, Ezh1-overexpressing, and Ezh2-overexpressing iMEFs untreated or treated with OHT to remove endogenous Ezh2 expression. Specific antibodies are indicated at the *left*. (*F*) Heat map representing the mean-centered expression of Ezh2-responsive targets in wild-type, Ezh2^Δ/Δ^, and Ezh2^Δ/Δ^ iMEFs overexpressing either Ezh2 or Ezh1. (*G*) H3K27me3 density plot around the TSSs of genes for which the absence of endogenous Ezh2 is rescued by Ezh1 (blue line) or not (red line). Analyses presented in *C*, *D*, *F*, and *G* were performed using measurements on two biological replicates.

Our analysis suggests that partial impairment of PRC2 might be sufficient to promote tumorigenesis. We therefore analyzed how incomplete disruption of PRC2 affects transcription of Polycomb target genes. For this purpose, we used a c-Myc transformed, Ezh2 conditional mouse embryonic fibroblast (iMEF) clonal cell line. This model allows OHT-dependent deletion of *Ezh2* and results in a drastic reduction of H3K27me3 and subsequent up-regulation of a small cohort of H3K27me3-positive genes, which we refer to as direct responsive targets (Fig. 5A). To assess the impact of a milder down-regulation of H3K27me3, we analyzed gene expression at an early time point after OHT treatment such that the mark was only partially depleted (day 5 after OHT treatment) (Fig. 5B). Only a subset (9%) of responsive genes was up-regulated at this time point (Fig. 5C; Supplemental Fig. S5A). Strikingly, early responsive genes were characterized by a low level of H3K27me3 specifically in the promoter region as compared with late responsive genes (Fig. 5D). This result suggests that accumulation of the mark in the promoter region controls the robustness of transcriptional repression.

To confirm that partial loss of H3K27me3 indeed releases the silencing of a subset of PRC2 target genes, we performed a complementary experiment in which we rescued the loss of Ezh2 by Ezh1, an enzyme that was previously reported to have a reduced enzymatic activity relative to Ezh2 (Margueron et al. 2008). In this experiment, Ezh1 or Ezh2 was stably expressed in cells before OHT-induced deletion of endogenous *Ezh2*. As expected, the global level of H3K27me3 was significantly lower in the Ezh1 rescue condition than in the control Ezh2 rescue condition (Fig. 5E, cf. lanes 5 and 6), and the genomic distribution of H3K27me3 was uniformly weaker in the Ezh1 rescue condition (Supplemental Fig. S5B). Responsive genes that could not be rescued by Ezh1 (26%) (Fig. 5F) had an initial lower enrichment of the mark in their promoter region compared with genes for which expression was rescued (Fig. 5G), thus corroborating our time-course analysis (Fig. 5D). Altogether, these results indicate that H3K27me3 accumulation in the promoter region is linked to robustness toward depletion of the mark; a mild decrease of H3K27me3 selectively impairs silencing of genes that have a low level of the mark in their promoter.

### Impaired PRC2 function leads to transcriptional instability

**Figure 6.**
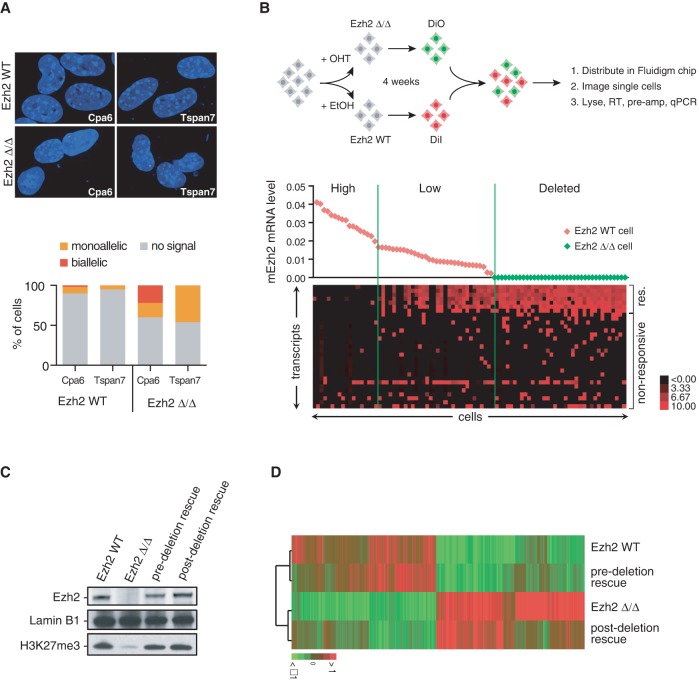
Impaired PRC2 function leads to transcriptional instability. (*A*) Nascent RNA FISH analysis of two responsive Ezh2 targets in Ezh2 wild-type and Ezh2 mutant iMEFs. The *Cpa6* gene is autosomal, while the *Tspan7* gene is localized on the X chromosome and thus only presents in one copy in this male cell line. The *top* panel shows representative examples of RNA FISH signals, and the *bottom* graph shows relative proportions of nuclei with no signal, one pinpoint (monoallelic), and two pinpoints (biallelic) over a minimum of 50 nuclei. (*B*, *top*) Experimental scheme for the single-cell analysis of PRC2 target genes. (*Bottom*) Single-cell analysis of the *Ezh2* transcript and selected responsive (res.) and nonresponsive genes. Forty-nine Ezh2 wild-type and 37 Ezh2 mutant cells were analyzed by RT-qPCR on a Biomark-HD system. *Ezh2* mRNA level in individual cells is plotted at the *top*; red diamonds represent Ezh2 wild-type cells (DiI-positive), and green diamonds indicate Ezh2^Δ/Δ^ cells (DiO-positive). A heat map representing the mean-centered, log_2_ transformed expression of selected target genes is displayed at the *bottom*. (*C*) Western blot of Ezh2, H3K27me3, and Lamin B1 as a loading control in different conditions as indicated at the *top* of each lane. (*D*) Heat map showing hierarchical clustering of transcripts in Ezh2 wild-type, Ezh2^Δ/Δ^, and pre- and post-deletion rescue conditions.

Alterations of PRC2 have been observed in cancers of different origin, indicating a fundamental, tissue-independent role in tumor suppression. However, disruption of PRC2 only results in the detectable up-regulation of a minority of tissue-specific genomic targets (Fig. 5A; Ezhkova et al. 2009; Woodhouse et al. 2013). We reasoned that, in addition, low-frequency (e.g., stochastic) responses might occur at the level of nonresponsive targets, leading to increased transcriptional instability. We thus asked how responsive and nonresponsive targets would be expressed in the presence or absence of Ezh2 at the level of individual cells. RNA FISH analysis indicated that responsive targets are expressed at low frequency in the presence of the enzyme (Fig. 6A). Single-cell RT-qPCR confirmed this observation and revealed that responsive target genes are detected in a subset of Ezh2 wild-type cells expressing a low level of the enzyme (Fig. 6B). Interestingly, while some nonresponsive genes were insensitive to Ezh2 status (Fig. 6B, bottom genes), a number of genes became activated in *Ezh2*-low cells in a sparse fashion (Fig. 6B, middle genes). Deletion of *Ezh2* resulted in a full derepression of responsive genes, while the frequency of expression was increased for nonresponsive genes. Remarkably, while responsive targets were expressed in a concerted—i.e., deterministic—fashion (e.g., cells expressed either all or none of the responsive targets), the expression of nonresponsive targets seemed probabilistic, with each cell expressing a different combination of genes. Since Polycomb target genes represent on the order of 3000–4000 genes, the observed effects of PRC2 disruption are expected to translate into widespread transcriptional instability. Thus, gene expression analysis at the single-cell level reveals that changes occurring at the level of Polycomb target genes are much more profound than previously appreciated.

We next asked whether disruption of PRC2 would translate into a long-lasting impact on gene expression. We therefore inquired whether reintroduction of Ezh2 in Ezh2 knockout iMEFs could revert loss of gene silencing. We compared Ezh2 wild-type, Ezh2 knockout, and Ezh2 rescue before and after deletion of the endogenous gene (hereafter called predeletion and post-deletion rescues). In both pre- and post-deletion rescue conditions, the global levels of Ezh2 and associated H3K27me3 were similar to that of wild-type cells (Fig. 6C). Strikingly, although the predeletion rescue prevented most transcriptional changes resulting from the absence of endogenous Ezh2, the post-deletion rescue failed to revert the transcriptional status of the majority of transcripts and clustered with the Ezh2 knockout condition (Fig. 6D). This indicates that transient disruption of PRC2 results in a permanent epigenetic switch in gene expression.

Thus, PRC2 safeguards genome-wide silencing through fine-tuned H3K27me3. Perturbation of this equilibrium results in both predictable, deterministic responses and stochastic loss of gene silencing with irreversible consequences on gene expression programs.

## Discussion

The current paradigm concerning the role of EZH2 in solid tumors postulates that abnormally high levels of this enzyme contribute to malignant transformation. Our study challenges this hypothesis. We present evidence indicating that high expression of EZH2 is a consequence rather than a cause of cancer and that, in breast cancer, disruption of the PRC2 machinery is likely to promote tumor development.

Using two mouse models of prostate cancer and one model of mammary tumorigenesis, we found that EZH2, although highly up-regulated in cancerous tissue, is dispensable for tumor progression. The strong correlation of EZH2 levels with proliferation markers in transcriptome analyses and in our tumorgraft series suggest that high EZH2 expression in cancers is predominantly a consequence of increased cell proliferation rate. Through fine-tuning of Ezh2 expression in vitro, we demonstrate that the tight coupling of Ezh2 expression to proliferation is required to oppose cell division-mediated dilution of H3K27me3, as previously hypothesized (Hansen et al. 2008). This is evidenced by the failure to maintain H3K27me3 with rising proliferation rates when the increase of EZH2 is not sufficient. These data imply that relative levels compared with the proliferation rate rather than absolute levels of EZH2 are a key factor in determining H3K27me3 levels. It also suggests that anti-correlated levels of the enzyme and the mark can be caused by a relative reduction of PRC2 activity compared with proliferation. Our analysis of EZH2 and H3K27me3 levels in our PDX series indeed suggests that this is likely to be the case, since proliferation, although positively influencing EZH2 levels, negatively modulates H3K27me3 abundance. Thus, PRC2 activity might not be sufficient to maintain the mark in rapidly dividing breast cancer cells. We propose that these observations reconcile contradictory data reporting inverse variations of EZH2 and H3K27me3 in several tumor types (Wei et al. 2008; Holm et al. 2012; Xu et al. 2012; Healey et al. 2014; Bae et al. 2015).

In addition, we found that while high *EZH2* expression is overall correlated to a poor prognosis in breast cancer, this association can be subdivided into two opposite components. The first component, originating from the coupling of *EZH2* expression to proliferation, associates high *EZH2* with an adverse outcome. However, we found that copy number-driven, proliferation-independent expression of *EZH2* displays an inverse association with tumor outcome, with low expression of *EZH2* being linked to a poor prognosis. This finding emphasizes the need to carefully account for the effect of proliferation when assessing the prognostic value of a given marker in cancers. In addition, this result suggests that decreased levels of EZH2 relative to proliferation might accelerate tumor development. In support of a protective role for PRC2 in breast cancer, we found that mutations in PRC2 core components are associated with a poor prognosis and documented several mutations in *EZH2* and PRC2 core component *SUZ12* in breast cancer metastases. One mutation in *EZH2* is predicted to profoundly affect its function. Finally, inactivation of *EZH2* in a prototypical human breast cancer cell line promotes in vitro invasion and in vivo tumor growth. Together, our findings indicate that EZH2 is likely to constrain breast tumorigenesis.

Although several studies have assessed the role of EZH2 in prostate and breast tumorigenesis, our study is, to our knowledge, the first to use genetic tools in both mouse and humans models. Of note, a recent study investigating the role of Ezh2 in a Brca1 deficiency-based model of mammary tumorigenesis found that deletion of *Ezh2* shortens the latency of tumor formation (Bae et al. 2015), further reinforcing the view that the enzyme might inhibit breast tumorigenesis.

In addition to leukemia and MPNST, a tumor-suppressive role for PRC2 has been suggested in a mouse model of pancreatic cancer (Mallen-St Clair et al. 2012) and in renal cancer (Vanharanta et al. 2013). Moreover, in pediatric glioblastomas, point mutations resulting in a change from lysine to methionine at position 27 of histone H3 (H3K27M) have been shown to inhibit PRC2 activity (Lewis et al. 2013), suggesting that disruption of the Polycomb machinery might be a recurring theme in cancers. However, how PRC2 impairment is linked to tumor progression is currently unclear. Our transcriptomic analysis revealed that alterations of PRC2 activity result in both a deterministic activation of a subset of PRC2 target genes and a broader stochastic activation of gene expression. While the former is expected to control the immediate biological response to Ezh2 inhibition, the latter, by increasing the plasticity of gene expression programs, might lead to long-term responses. Such a distinction between early and late response to Ezh2 inhibition was recently reported in a model of glioblastoma in which prolonged knockdown of Ezh2 results in the emergence of “escaper” tumors characterized by an aggressive phenotype (de Vries et al. 2015). It is tempting to speculate that the transcriptional instability of Polycomb targets as a consequence of Ezh2 knockdown might have fueled the emergence of escaper tumors. Given the high mutation rates reported for other chromatin regulators in cancer, it will be interesting to determine whether they also results in increased transcriptomic instability.

Several EZH2 inhibitors are entering clinical trials. It is expected that tumor types in which EZH2 gain-of-function mutations occur (e.g., DLBCL and FL) (Campbell et al. 2015) as well as tumors harboring mutations in SWI/SNF components (Wilson et al. 2010; Knutson et al. 2013; Bitler et al. 2015) might benefit from these molecules. However, the long-term impact of such inhibition should be carefully examined in light of transcriptional instability and irreversible changes resulting from PRC2 disruption.

Finally, our analysis prompts a careful examination of the contribution to tumorigenesis of genes whose expression is linked to proliferation. We propose that applying a similar analysis to other proliferation-associated genes, including key players of epigenetic modifications, could help clarify their contribution to cancer.

## Materials and methods

### Plasmids

The MSCVhygro-Flag-Ezh2 retroviral vector was obtained from Addgene (24926). MSCVhygro-Flag-Ezh1 was generated by subcloning. Following retroviral infection, cells were selected with 400 µg/mL Hygromycin B (Life Technologies). pLKO.1-shEzh2 was purchased from Dharmacon (clone ID TRCN0000039042; antisense sequence TTTCTTTCAGTTCTTCTGCGG). Oligonucleotides corresponding to a scramble shRNA (antisense sequence CGAGGGCGACTTAACCTTAGG) were cloned into the pLKO.1 vector. Following lentiviral infection, cells were selected with 2 µg/µL puromycin (Life Technologies).

### Cell lines

iMEF cells were grown in DMEM supplemented with 10% FCS, 100 mM nonessential amino acids, 1 mM L-glutamine. *Ezh2*^*flox/flox*^;*ROSA26-CreERT2* or *Ezh2*^*flox*/Δ^;*ROSA26-CreERT2* MEF cells were isolated from 13.5-d-old embryos and subsequently infected with the following retroviral constructs: pMXs-hc-MYC (Addgene, 17220) to generate c-Myc iMEFs, pBABE-hygro p53 DD (Addgene, 9058) to generate p53-DN iMEFs, and Ndy1-MigR1 (kindly provided by Philip N. Tsichlis) to generate Ndy1 iMEFs. A clone was obtained by limiting dilution of a pool of c-Myc *Ezh2*^*flox*/Δ^*;ROSA26-CreERT2* iMEFs. For conditional deletion of *Ezh2*, cells were treated with 4-hydroxytamoxifen (Sigma) at a final concentration ranging from 1 nM to 1 µM. For Ezh1 and Ezh2 rescue experiments, cells were infected with MSCVhygro-Flag-Ezh1 or MSCVhygro-Flag-Ezh2 ecotropic retroviruses.

The Myc-CaP mouse prostate cancer cell line was generously provided by Charles L. Sawyers, and cells were grown in DMEM supplemented with 10% FCS, 100 mM nonessential amino acids, and 1 mM L-glutamine. To obtain optimal growth conditions, the growth medium was supplemented with a growth factor cocktail composed of 25 µg/mL bovine pituitary extract (Life Technologies), 5 µg/mL bovine insulin (Sigma), and 6 ng/mL recombinant human epidermal growth factor (Sigma).

### Cell growth assay

Twenty-thousand cells were plated in six-well dishes in triplicates and counted every 24 h over 4 d using a Vi cells counter (Beckman-coulter).

### Mice

All animals used in the studies were handled with care, and experiments were done according to the guidelines from French legislation (Pten and N1ic models) or U.S. legislation (Hi-Myc model) and institutional policies. For the prostate cancer study, *PB4-Cre* (kindly provided by M. Chen), *Pten*^*flox*/+^ (kindly provided by O. Lantz), *Hi-Myc* (Jackson laboratory), and *Ezh2*^*flox*/+^ (generously provided by A. Tarakhovsky) mice were used. For the breast cancer study, *MMTV-Cre*, *Rosa-N1ic*, and *Ezh2*^*flox*/+^ mice were used.

For xenografting experiments, 6-wk-old female CB17-SCID mice were purchased from Janvier Laboratories. MDA-MB-231 cells (1 × 10^6^) were orthotopically injected into the mammary fat pads of 20 mice. Control cells (one of three alleles of *EZH2* mutated) were grafted on the left side of each mouse, and EZH2^−^ cells were grafted on the right side of each mouse. Mice were sacrificed 10 wk after injection, and the resulting tumors were measured and collected.

### Constitutive knockout of *EZH2* in the MDA-MB-231 cell line

Mutation of all three alleles of *EZH2* in the MDA-MB-231 cell line was performed using CRISPR/CAS9 technology. A donor template encoding a puromycin selection cassette was nucleofected at a 1:1:1 ratio with CAS9 (Addgene, 41815) and *EZH2*-specific guide RNA (gRNA; build from gRNA cloning vector; Addgene, 41824). Homology-directed repair of the double-strand break resulted in the insertion of an FRT-flanked puromycin resistance gene in-frame with the first exon of EZH2. The first allele of *EZH2* was targeted using the following guide sequence: GTATACCTAATTCCTGTAAT. Sequencing of the remaining alleles revealed a single nucleotide insertion at the target site. Consequently, after flippase-mediated excision of the puromycin resistance cassette from the first allele, the remaining alleles were targeted using the following guide sequence: GTATACCTAATTCCTGTTAA. The resulting clone was thus used as a constitutive *EZH2* knockout, using the parental clone (one allele targeted) as a control in all experiments.

### Inhibition of EZH1 and EZH2

Inhibition of EZH1 and EZH2 was achieved by a treatment with 1 µM UNC1999 (Sigma).

### Formation of the spheroids and type I collagen invasion assay

Multicellular spheroids of MDA-MB-231 cells were prepared using the hanging droplet method (Kelm et al. 2003), with 3 × 10^3^ cells in 20-µL droplets in complete L15 medium + 1% volume of collagen I for 3 d. Next, spheroids were embedded in 2.2 mg/mL type I collagen gel (T0) prepared from acid extract of rat tail tendon (from BD Biosciences) and incubated for 2 d (T2).

### Imaging and quantification of the area of invasion

Samples were fixed at T0 and T2 and costained with fluorescent phalloidin to label F-actin and DAPI. Images were taken with a confocal LSM 510 (Zeiss) microscope with a 5× dry objective, collecting stacks of images along the *Z*-axis with 10-µm intervals between optical sections.

Quantification of invasion was done with ImageJ software (http://rsb.info.nih.gov/ij) by estimating the diameter of spheroids at T0 and T2 as described (Rey et al. 2011). These values were averaged and used to calculate the mean invasion area (π*r*^2^). The mean invasion area at T2 was normalized to the mean invasion area at T0.

### Antibodies

Antibodies against Ezh1, Ezh2, Eed, Suz12, and H3K27me2/3 (Western blot and ChIP [chromatin immunoprecipitation]/ChIP-seq [ChIP combined with deep sequencing]) were previously described (Margueron et al. 2008); total H3 (39163) and H3K27me3 (39155) for ChIP-seq were purchased from Active Motif; Lamin B1 (ab16048) was purchased from Abcam; Ezh2 (NCL-L-EZH2) for IHC on PDXs was purchased from Novocastra; H3K27me3 (C36B11) for IHC on PDXs was purchased from Cell Signaling; Flag M2 was purchased from Sigma (F1804); Nkx3.1 antibody was a generous gift from Dr C. Abate-Shen; SMA antibody was purchased from Dako; PCNA, AR, and Sirt1 antibodies were purchased from Santa Cruz Biotechnology; and tubulin antibody was purchased from Sigma.

### Nuclear extracts

For nuclear extract preparation, cells were incubated with buffer A (10 mM Hepes at pH 7.9, 2.5 mM MgCl_2_, 0.25 M sucrose, 0.1% NP40, 0.5 mM DTT, 1 mM PSMF) for 10 min on ice, centrifuged at 8000 rpm for 10 min, resuspended in buffer B (25 mM Hepes at pH 7.9, 1.5 mM MgCl_2_, 700 mM NaCl, 0.5 mM DTT, 0.1 mM EDTA, 20% glycerol), sonicated, and centrifuged at 14,000 rpm for 15 min.

### Western blot quantification

Image acquisition of Western blots was performed on a LAS 4000 imager (Leica), and signal intensity was measured using ImageJ software.

### Tissue extracts

Protein extracts from tissues were prepared as previously described (Margueron et al. 2008).

### RT-qPCR

Total RNA was isolated using the RNeasy minikit (Qiagen). cDNA was synthetized using the High-Capacity cDNA RT kit (Applied Biosystems, 4368814), and qPCR was performed with technical triplicate using SYBR Green reagent (Roche) on ViiA7 equipment (Applied Biosystems). At least three independent biological experiments were performed for each assay, and negative control RTs were always included. Primers sequences are in Supplemental Table S3.

### IHC

IHC on mouse tissue was performed as previously described (Margueron et al. 2008).

IHC analysis on PDXs was performed on tissue microarrays obtained from treated xenografts as described in Cottu et al. (2014). Samples were dewaxed, and antigen retrieval was performed for 20 min in pH 6 citrate buffer. Developing was performed with the “Bond refine detection” kit (Leica biosystems); samples were incubated with diaminobenzidine for 7 min followed by counterstaining with hematoxylin for 4 min.

### IHC quantification

IHC images were first processed with the ImageJ Colour Deconvolution plug-in (http://www.mecourse.com/landinig/software/cdeconv/cdeconv.html) in order to separate HE and DAB signals. For each tissue microarray (TMA), signal intensity of DAB signal over HE signal was then calculated. Alternatively, when the level of background was too high (i.e., for EZH2, showing areas of nonspecific staining), staining intensity was scored on a scale of 0 to 3 in a blind fashion. Importantly, both software-assisted and visual scoring yielded highly correlated results.

### ChIP

ChIPs were performed as described previously (Margueron et al. 2008). Cell confluence and amount of starting material were kept constant by plating a defined number of cells the day before cross-linking. Quantification was done as described for the RT-qPCR. Primers sequences are in Supplemental Table S3.

### ChIP-seq

ChIP was performed as described above, starting from 25 µg of chromatin; magnetic Dynabeads coupled to Protein A were used for the immunoprecipitation (Invitrogen). Incubation with micrococcal nuclease was performed to obtain mostly mononucleosome-sized fragments (150 base pairs [bp]). Libraries were prepared according to the manufacturer's instructions (TruSeq ChIP sample preparation kit, Illumina). High-throughput sequencing was performed on a SOLiD 5500 and an Illumina Hi-Seq 2500. Single-end 75-bp (SOLiD) or 100-bp (Illumina) reads were mapped on the mouse reference genome (mm9) using Bowtie 2 (version 2.1.0) (Langmead and Salzberg 2012), allowing one mismatch in the seed (22 bp) and reporting one location in case of multiple mapping hits. PCR duplicates were then removed using PicardTools (version 1.65; http://picard.sourceforge.net).

### ChIP-seq analysis

Chip-seq data for H3K27me3 were generated in duplicates by the next-generation sequencing (NGS) platform at the Institut Curie (T. Rio Frio). From ∼50 million SOLiD 5500 75-bp reads per sample, 50% were uniquely mapped (minimum mapQ = 10) onto the mouse reference genome (mm9) using Bowtie (parameters: -S -C -p 8 -q -y --col-keepends -l 28 -n 2 -e 70 -a --best --strata -m 1). From 50 million Illumina HiSeq 2500 100-bp reads per sample, ∼98% were uniquely mapped onto the mouse reference genome (mm9) using Bowtie 2, allowing one mismatch in the seed (22 bp long). Given the high proportion of mapped read duplicates observed (until 22%), the duplicates were removed using the rmdup function of SAMTools. The mapped read data were then normalized to the total number of reads (25 million) by performing a down-sampling with Picard, and a coefficient factor was applied in some conditions (Ezh2^−/−^: 0.05; Ezh1 rescue: 0.25) according to the amount of immunoprecipitated DNA obtained during the ChIP preparation. Peak calling for each sample was done with MACS 2 (2.0.10) with default parameters for histone analysis by specifying a fragment size of 150 bp and the input DNA as control. From the significant peak regions, the differential binding analysis was performed with the R package DiffBind. Only reproducible peaks between replicates were kept for read counting. Significant differential peaks were identified with a maximum false discovery rate (FDR) of 1% and a minimum value of fold change equal to 2 for each comparison. Peaks were quantified using HOMER.

### Transcriptome data analysis

Microarray data were generated in duplicates by the microarray core facility of the Institut Curie (D. Gentien) using Affymetrix Mouse Gene 1.1 ST arrays (targeting 21041 genes). Raw data were normalized with the Robust Multiarray Average (RMA) method available in the Bioconductor R package oligo and the “pd.mogene.1.1.st.v1” annotation package. For rescue experiments, given the observed batch bias between the first and second replicates, the data were then batch-corrected using a linear model (Limma R package). Differential gene expression analysis was done using the RankProduct R package, and significantly underexpressed or overexpressed genes were identified with a minimum adjusted *P*-value of 15% and a minimum value of fold change equal to 2. Hierarchical clustering analysis was performed using Cluster 3.0, and heat maps were generated with TreeView.

### Gene expression, copy number, and survival analysis of breast primary tumors

This study makes use of data generated by METABRIC (first described in Curtis et al. (2012). Funding for the project was provided by Cancer Research UK and the British Columbia Cancer Agency Branch. Upon access request, single-nucleotide polymorphism (SNP) 6.0 copy number and Illumina HT-12 expression data for nearly 2000 primary breast tumors were available through the European Genome–Phenome Archive (http://www.ebi.ac.uk/ega) under accession number EGAS00000000083.

For survival analyses, disease-specific survival was used as the end point. Follow-up time was defined as time from diagnosis until death from breast cancer or time of last follow-up if the patient was not known to have died. Kaplan-Meier and ROC analyses were performed using Prism 6. Statistical significance was evaluated with the log-rank test. The hazard ratio of the highest to the lowest survival rate was calculated using the log-rank method. For survival analysis on all genes located on chromosome 7, the univariate Cox model was applied to each gene by comparing the outcome linked to tumors with genetic loss with that of the remaining samples. A FDR was used to correct for multiple testing.

### Targeted sequencing of PRC2 genes in breast cancer metastases

The coding sequences of the EED, EZH2, and SUZ12 genes were analyzed using a targeted NGS approach. Experiments were performed on the NGS platform of the Cochin Hospital, Paris (Assistance Publique, Hopitaux de Paris, France). Briefly, the custom primer panel targeting the three genes (coding exons and IVS boundaries) was designed using AmpliSeq Designer (Life Technologies). For NGS library preparation, the Ion AmpliSeq 2.0 library kit was used according to the manufacturer's instructions. Amplified libraries were purified using Agencourt AMPure XP beads (Beckman Coulter). Prior to library pooling and sequencing sample preparation, amplified libraries were quantified using the Qubit fluorometer system (Agilent Technologies). Emulsion PCR and enrichment were performed on the Ion OneTouch and Ion OneTouch ES instruments with the Ion PGM template OT2 400 and Ion PGM sequencing 400 kits (Life Technologies). The template-positive ion sphere particles were loaded on Ion 318 chips and sequenced with an Ion PGM system (Life Technologies). Sequence alignment and extraction of SNPs and short insertions/deletions were performed using the Variant Caller plug-in on Ion Torrent suite version 4.4 and Ion Reporter version 4.4 (Life Technologies). DNA sequences were visualized using the Integrated Genomics Viewer (version 2.3.3) from the Broad Institute.

### RNA FISH

RNA FISH was performed as described elsewhere (Chaumeil et al. 2008). The following BAC probes (CHORI) were used: RP23-333D4 (*Cpa6*) and RP23-40H14 (*Tspan7*).

### Single-cell RT-qPCR analysis

To discriminate OHT and vehicle-treated Ezh2-conditional iMEFs, DiI (vehicle-treated) or DiO (OHT-treated) was added in growth medium for 4 h. Cells were then trypsinized and counted on a Vi cells counter (Beckman-Coulter). The average diameter of iMEFs was 13 μm. After mixing OHT- and vehicle-treated cells in equal proportions, 250,000 cells per milliliter were mixed at a 3:2 ratio in C1 cell suspension reagent (Fluidigm) before being loaded on a primed C1 Single-Cell Auto Prep Integrated Fluidic Circuit (Fluidigm). Cells were then visualized under an inverted fluorescent microscope (Leica) to assess viability and assignment of red (OHT-treated) and green (vehicle-treated) cells. Lysis, RT, and preamplifications were performed according to the manufacturer's protocol using Ambion Single Cell-to-CT kit (LifeTechnologies). Preamplified cDNA was analyzed by high-throughput qPCR on a Biomark-HD system (Fluidigm). The complete list of primers is in Supplemental Table S4. A qPCR primer pair designed on the region of the set domain that is deleted upon OHT treatment served as an independent genotype assignment and was found to closely match the color assignment. Only single cells with matching genotype/color assignments were considered for analysis.

### Data access

All ChIP-seq data sets have been deposited in the Gene Expression Omnibus repository (GSE59427).

## Supplementary Material

Supplemental Material

## References

[WASSEFGAD269522C1] Bachmann IM, Halvorsen OJ, Collett K, Stefansson IM, Straume O, Haukaas SA, Salvesen HB, Otte AP, Akslen LA. 2006 EZH2 expression is associated with high proliferation rate and aggressive tumor subgroups in cutaneous melanoma and cancers of the endometrium, prostate, and breast. J Clin Oncol 24: 268–273. 16330673 10.1200/JCO.2005.01.5180

[WASSEFGAD269522C2] Bae WK, Yoo KH, Lee JS, Kim Y, Chung IJ, Park MH, Yoon JH, Furth PA, Hennighausen L. 2015 The methyltransferase EZH2 is not required for mammary cancer development, although high EZH2 and low H3K27me3 correlate with poor prognosis of ER-positive breast cancers. Mol Carcinog 54: 1172–1180. 25043748 10.1002/mc.22188 PMC4286524

[WASSEFGAD269522C3] Benetatos L, Vartholomatos G, Hatzimichael E. 2014 Polycomb group proteins and MYC: the cancer connection. Cell Mol Life Sci 71: 257–269. 23897499 10.1007/s00018-013-1426-x PMC11113285

[WASSEFGAD269522C4] Bitler BG, Aird KM, Garipov A, Li H, Amatangelo M, Kossenkov AV, Schultz DC, Liu Q, Shih IeM, Conejo-Garcia JR, 2015 Synthetic lethality by targeting EZH2 methyltransferase activity in ARID1A-mutated cancers. Nat Med 21: 231–238. 25686104 10.1038/nm.3799 PMC4352133

[WASSEFGAD269522C5] Bolos V, Mira E, Martinez-Poveda B, Luxan G, Canamero M, Martinez AC, Manes S, de la Pompa JL. 2013 Notch activation stimulates migration of breast cancer cells and promotes tumor growth. Breast Cancer Res 15: R54. 23826634 10.1186/bcr3447 PMC3978930

[WASSEFGAD269522C7] Bracken AP, Helin K. 2009 Polycomb group proteins: navigators of lineage pathways led astray in cancer. Nat Rev Cancer 9: 773–784. 19851313 10.1038/nrc2736

[WASSEFGAD269522C8] Bracken AP, Pasini D, Capra M, Prosperini E, Colli E, Helin K. 2003 EZH2 is downstream of the pRB–E2F pathway, essential for proliferation and amplified in cancer. EMBO J 22: 5323–5335. 14532106 10.1093/emboj/cdg542 PMC213796

[WASSEFGAD269522C9] Bracken AP, Kleine-Kohlbrecher D, Dietrich N, Pasini D, Gargiulo G, Beekman C, Theilgaard-Monch K, Minucci S, Porse BT, Marine JC, 2007 The Polycomb group proteins bind throughout the INK4A-ARF locus and are disassociated in senescent cells. Genes Dev 21: 525–530. 17344414 10.1101/gad.415507 PMC1820894

[WASSEFGAD269522C10] Campbell JE, Kuntz KW, Knutson SK, Warholic NM, Keilhack H, Wigle TJ, Raimondi A, Klaus CR, Rioux N, Yokoi A, 2015 EPZ011989, a potent, orally-available EZH2 inhibitor with robust in vivo activity. ACS Med Chem Lett 6: 491–495. 26005520 10.1021/acsmedchemlett.5b00037 PMC4434464

[WASSEFGAD269522C11] Chase A, Cross NC. 2011 Aberrations of EZH2 in cancer. Clin Cancer Res 17: 2613–2618. 21367748 10.1158/1078-0432.CCR-10-2156

[WASSEFGAD269522C12] Chaumeil J, Augui S, Chow JC, Heard E. 2008 Combined immunofluorescence, RNA fluorescent in situ hybridization, and DNA fluorescent in situ hybridization to study chromatin changes, transcriptional activity, nuclear organization, and X-chromosome inactivation. Methods Mol Biol 463: 297–308. 18951174 10.1007/978-1-59745-406-3_18

[WASSEFGAD269522C13] Cottu P, Bieche I, Assayag F, El Botty R, Chateau-Joubert S, Thuleau A, Bagarre T, Albaud B, Rapinat A, Gentien D, 2014 Acquired resistance to endocrine treatments is associated with tumor-specific molecular changes in patient-derived luminal breast cancer xenografts. Clin Cancer Res 20: 4314–4325. 24947930 10.1158/1078-0432.CCR-13-3230

[WASSEFGAD269522C14] Curtis C, Shah SP, Chin SF, Turashvili G, Rueda OM, Dunning MJ, Speed D, Lynch AG, Samarajiwa S, Yuan Y, 2012 The genomic and transcriptomic architecture of 2,000 breast tumours reveals novel subgroups. Nature 486: 346–352. 22522925 10.1038/nature10983 PMC3440846

[WASSEFGAD269522C15] De Raedt T, Beert E, Pasmant E, Luscan A, Brems H, Ortonne N, Helin K, Hornick JL, Mautner V, Kehrer-Sawatzki H, 2014 PRC2 loss amplifies Ras-driven transcription and confers sensitivity to BRD4-based therapies. Nature 514: 247–251. 25119042 10.1038/nature13561

[WASSEFGAD269522C16] de Vries NA, Hulsman D, Akhtar W, de Jong J, Miles DC, Blom M, van Tellingen O, Jonkers J, van Lohuizen M. 2015 Prolonged Ezh2 depletion in glioblastoma causes a robust switch in cell fate resulting in tumor progression. Cell Rep 10: 383–397. 10.1016/j.celrep.2014.12.028 25600873

[WASSEFGAD269522C17] Ellwood-Yen K, Graeber TG, Wongvipat J, Iruela-Arispe ML, Zhang J, Matusik R, Thomas GV, Sawyers CL. 2003 Myc-driven murine prostate cancer shares molecular features with human prostate tumors. Cancer Cell 4: 223–238. 14522256 10.1016/s1535-6108(03)00197-1

[WASSEFGAD269522C18] Ezhkova E, Pasolli HA, Parker JS, Stokes N, Su IH, Hannon G, Tarakhovsky A, Fuchs E. 2009 Ezh2 orchestrates gene expression for the stepwise differentiation of tissue-specific stem cells. Cell 136: 1122–1135. 19303854 10.1016/j.cell.2008.12.043 PMC2716120

[WASSEFGAD269522C19] Gonzalez ME, Li X, Toy K, DuPrie M, Ventura AC, Banerjee M, Ljungman M, Merajver SD, Kleer CG. 2009 Downregulation of EZH2 decreases growth of estrogen receptor-negative invasive breast carcinoma and requires BRCA1. Oncogene 28: 843–853. 19079346 10.1038/onc.2008.433 PMC2643353

[WASSEFGAD269522C20] Hansen KH, Bracken AP, Pasini D, Dietrich N, Gehani SS, Monrad A, Rappsilber J, Lerdrup M, Helin K. 2008 A model for transmission of the H3K27me3 epigenetic mark. Nat Cell Biol 10: 1291–1300. 18931660 10.1038/ncb1787

[WASSEFGAD269522C21] Healey MA, Hu R, Beck AH, Collins LC, Schnitt SJ, Tamimi RM, Hazra A. 2014 Association of H3K9me3 and H3K27me3 repressive histone marks with breast cancer subtypes in the Nurses’ Health Study. Breast Cancer Res Treat 147: 639–651. 25224916 10.1007/s10549-014-3089-1

[WASSEFGAD269522C22] Holm K, Grabau D, Lovgren K, Aradottir S, Gruvberger-Saal S, Howlin J, Saal LH, Ethier SP, Bendahl PO, Stal O, 2012 Global H3K27 trimethylation and EZH2 abundance in breast tumor subtypes. Mol Oncol 6: 494–506. 22766277 10.1016/j.molonc.2012.06.002 PMC5528390

[WASSEFGAD269522C23] Kelm JM, Timmins NE, Brown CJ, Fussenegger M, Nielsen LK. 2003 Method for generation of homogeneous multicellular tumor spheroids applicable to a wide variety of cell types. Biotechnol Bioeng 83: 173–180. 12768623 10.1002/bit.10655

[WASSEFGAD269522C24] Kleer CG, Cao Q, Varambally S, Shen R, Ota I, Tomlins SA, Ghosh D, Sewalt RG, Otte AP, Hayes DF, 2003 EZH2 is a marker of aggressive breast cancer and promotes neoplastic transformation of breast epithelial cells. Proc Natl Acad Sci 100: 11606–11611. 14500907 10.1073/pnas.1933744100 PMC208805

[WASSEFGAD269522C25] Knutson SK, Warholic NM, Wigle TJ, Klaus CR, Allain CJ, Raimondi A, Porter Scott M, Chesworth R, Moyer MP, Copeland RA, 2013 Durable tumor regression in genetically altered malignant rhabdoid tumors by inhibition of methyltransferase EZH2. Proc Natl Acad Sci 110: 7922–7927. 23620515 10.1073/pnas.1303800110 PMC3651445

[WASSEFGAD269522C26] Koh CM, Iwata T, Zheng Q, Bethel C, Yegnasubramanian S, De Marzo AM. 2011 Myc enforces overexpression of EZH2 in early prostatic neoplasia via transcriptional and post-transcriptional mechanisms. Oncotarget 2: 669–683. 21941025 10.18632/oncotarget.327 PMC3248223

[WASSEFGAD269522C27] Konze KD, Ma A, Li F, Barsyte-Lovejoy D, Parton T, Macnevin CJ, Liu F, Gao C, Huang XP, Kuznetsova E, 2013 An orally bioavailable chemical probe of the lysine methyltransferases EZH2 and EZH1. ACS Chem Biol 8: 1324–1334. 23614352 10.1021/cb400133j PMC3773059

[WASSEFGAD269522C28] Langmead B, Salzberg SL. 2012 Fast gapped-read alignment with Bowtie 2. Nat Methods 9: 357–359. 22388286 10.1038/nmeth.1923 PMC3322381

[WASSEFGAD269522C29] Lee ST, Li Z, Wu Z, Aau M, Guan P, Karuturi RK, Liou YC, Yu Q. 2011 Context-specific regulation of NF-κB target gene expression by EZH2 in breast cancers. Mol cell 43: 798–810. 21884980 10.1016/j.molcel.2011.08.011

[WASSEFGAD269522C30] Le Tourneau C, Paoletti X, Servant N, Bieche I, Gentien D, Rio Frio T, Vincent-Salomon A, Servois V, Romejon J, Mariani O, 2014 Randomised proof-of-concept phase II trial comparing targeted therapy based on tumour molecular profiling vs conventional therapy in patients with refractory cancer: results of the feasibility part of the SHIVA trial. Br J Cancer 111: 17–24. 24762958 10.1038/bjc.2014.211 PMC4090722

[WASSEFGAD269522C31] Lewis PW, Muller MM, Koletsky MS, Cordero F, Lin S, Banaszynski LA, Garcia BA, Muir TW, Becher OJ, Allis CD. 2013 Inhibition of PRC2 activity by a gain-of-function H3 mutation found in pediatric glioblastoma. Science 340: 857–861. 23539183 10.1126/science.1232245 PMC3951439

[WASSEFGAD269522C32] Ma X, Ziel-van der Made AC, Autar B, van der Korput HA, Vermeij M, van Duijn P, Cleutjens KB, de Krijger R, Krimpenfort P, Berns A, 2005 Targeted biallelic inactivation of Pten in the mouse prostate leads to prostate cancer accompanied by increased epithelial cell proliferation but not by reduced apoptosis. Cancer Res 65: 5730–5739. 15994948 10.1158/0008-5472.CAN-04-4519

[WASSEFGAD269522C33] Maire V, Nemati F, Richardson M, Vincent-Salomon A, Tesson B, Rigaill G, Gravier E, Marty-Prouvost B, De Koning L, Lang G, 2013 Polo-like kinase 1: a potential therapeutic option in combination with conventional chemotherapy for the management of patients with triple-negative breast cancer. Cancer Res 73: 813–823. 23144294 10.1158/0008-5472.CAN-12-2633

[WASSEFGAD269522C34] Mallen-St Clair J, Soydaner-Azeloglu R, Lee KE, Taylor L, Livanos A, Pylayeva-Gupta Y, Miller G, Margueron R, Reinberg D, Bar-Sagi D. 2012 EZH2 couples pancreatic regeneration to neoplastic progression. Genes Dev 26: 439–444. 22391448 10.1101/gad.181800.111 PMC3305982

[WASSEFGAD269522C35] Margueron R, Li G, Sarma K, Blais A, Zavadil J, Woodcock CL, Dynlacht BD, Reinberg D. 2008 Ezh1 and Ezh2 maintain repressive chromatin through different mechanisms. Mol Cell 32: 503–518. 19026781 10.1016/j.molcel.2008.11.004 PMC3641558

[WASSEFGAD269522C36] Min J, Zaslavsky A, Fedele G, McLaughlin SK, Reczek EE, De Raedt T, Guney I, Strochlic DE, Macconaill LE, Beroukhim R, 2010 An oncogene–tumor suppressor cascade drives metastatic prostate cancer by coordinately activating Ras and nuclear factor-κB. Nat Med 16: 286–294. 20154697 10.1038/nm.2100 PMC2903662

[WASSEFGAD269522C37] Morin RD, Johnson NA, Severson TM, Mungall AJ, An J, Goya R, Paul JE, Boyle M, Woolcock BW, Kuchenbauer F, 2010 Somatic mutations altering EZH2 (Tyr641) in follicular and diffuse large B-cell lymphomas of germinal-center origin. Nat Genet 42: 181–185. 20081860 10.1038/ng.518 PMC2850970

[WASSEFGAD269522C38] Murtaugh LC, Stanger BZ, Kwan KM, Melton DA. 2003 Notch signaling controls multiple steps of pancreatic differentiation. Proc Natl Acad Sci 100: 14920–14925. 14657333 10.1073/pnas.2436557100 PMC299853

[WASSEFGAD269522C39] Nagalla S, Chou JW, Willingham MC, Ruiz J, Vaughn JP, Dubey P, Lash TL, Hamilton-Dutoit SJ, Bergh J, Sotiriou C, 2013 Interactions between immunity, proliferation and molecular subtype in breast cancer prognosis. Genome Biol 14: R34. 23618380 10.1186/gb-2013-14-4-r34 PMC3798758

[WASSEFGAD269522C40] Nikoloski G, Langemeijer SM, Kuiper RP, Knops R, Massop M, Tonnissen ER, van der Heijden A, Scheele TN, Vandenberghe P, de Witte T, 2010 Somatic mutations of the histone methyltransferase gene EZH2 in myelodysplastic syndromes. Nat Genet 42: 665–667. 20601954 10.1038/ng.620

[WASSEFGAD269522C41] Ntziachristos P, Tsirigos A, Van Vlierberghe P, Nedjic J, Trimarchi T, Flaherty MS, Ferres-Marco D, da Ros V, Tang Z, Siegle J, 2012 Genetic inactivation of the polycomb repressive complex 2 in T cell acute lymphoblastic leukemia. Nat Med 18: 298–301. 22237151 10.1038/nm.2651 PMC3274628

[WASSEFGAD269522C42] Rey M, Irondelle M, Waharte F, Lizarraga F, Chavrier P. 2011 HDAC6 is required for invadopodia activity and invasion by breast tumor cells. Eur J Cell Biol 90: 128–135. 20970878 10.1016/j.ejcb.2010.09.004

[WASSEFGAD269522C43] Seward S, Semaan A, Qazi AM, Gruzdyn OV, Chamala S, Bryant CC, Kumar S, Cameron D, Sethi S, Ali-Fehmi R, 2013 EZH2 blockade by RNA interference inhibits growth of ovarian cancer by facilitating re-expression of p21(waf1/cip1) and by inhibiting mutant p53. Cancer Lett 336: 53–60. 23603558 10.1016/j.canlet.2013.04.012

[WASSEFGAD269522C44] Simon JA, Kingston RE. 2013 Occupying chromatin: Polycomb mechanisms for getting to genomic targets, stopping transcriptional traffic, and staying put. Mol Cell 49: 808–824. 23473600 10.1016/j.molcel.2013.02.013 PMC3628831

[WASSEFGAD269522C45] Sneeringer CJ, Scott MP, Kuntz KW, Knutson SK, Pollock RM, Richon VM, Copeland RA. 2010 Coordinated activities of wild-type plus mutant EZH2 drive tumor-associated hypertrimethylation of lysine 27 on histone H3 (H3K27) in human B-cell lymphomas. Proc Natl Acad Sci 107: 20980–20985. 21078963 10.1073/pnas.1012525107 PMC3000297

[WASSEFGAD269522C46] Stylianou S, Clarke RB, Brennan K. 2006 Aberrant activation of notch signaling in human breast cancer. Cancer Res 66: 1517–1525. 16452208 10.1158/0008-5472.CAN-05-3054

[WASSEFGAD269522C47] Su IH, Basavaraj A, Krutchinsky AN, Hobert O, Ullrich A, Chait BT, Tarakhovsky A. 2003 Ezh2 controls B cell development through histone H3 methylation and Igh rearrangement. Nat Immunol 4: 124–131. 12496962 10.1038/ni876

[WASSEFGAD269522C48] Taylor BS, Schultz N, Hieronymus H, Gopalan A, Xiao Y, Carver BS, Arora VK, Kaushik P, Cerami E, Reva B, 2010 Integrative genomic profiling of human prostate cancer. Cancer Cell 18: 11–22. 20579941 10.1016/j.ccr.2010.05.026 PMC3198787

[WASSEFGAD269522C49] Tomlins SA, Mehra R, Rhodes DR, Cao X, Wang L, Dhanasekaran SM, Kalyana-Sundaram S, Wei JT, Rubin MA, Pienta KJ, 2007 Integrative molecular concept modeling of prostate cancer progression. Nat Genet 39: 41–51. 17173048 10.1038/ng1935

[WASSEFGAD269522C50] Vanharanta S, Shu W, Brenet F, Hakimi AA, Heguy A, Viale A, Reuter VE, Hsieh JJ, Scandura JM, Massague J. 2013 Epigenetic expansion of VHL–HIF signal output drives multiorgan metastasis in renal cancer. Nat Med 19: 50–56. 23223005 10.1038/nm.3029 PMC3540187

[WASSEFGAD269522C51] Varambally S, Dhanasekaran SM, Zhou M, Barrette TR, Kumar-Sinha C, Sanda MG, Ghosh D, Pienta KJ, Sewalt RG, Otte AP, 2002 The polycomb group protein EZH2 is involved in progression of prostate cancer. Nature 419: 624–629. 12374981 10.1038/nature01075

[WASSEFGAD269522C52] Varambally S, Cao Q, Mani RS, Shankar S, Wang X, Ateeq B, Laxman B, Cao X, Jing X, Ramnarayanan K, 2008 Genomic loss of microRNA-101 leads to overexpression of histone methyltransferase EZH2 in cancer. Science 322: 1695–1699. 19008416 10.1126/science.1165395 PMC2684823

[WASSEFGAD269522C53] Venet D, Dumont JE, Detours V. 2011 Most random gene expression signatures are significantly associated with breast cancer outcome. PLoS Comput Biol 7: e1002240. 22028643 10.1371/journal.pcbi.1002240 PMC3197658

[WASSEFGAD269522C54] Wang S, Gao J, Lei Q, Rozengurt N, Pritchard C, Jiao J, Thomas GV, Li G, Roy-Burman P, Nelson PS, 2003 Prostate-specific deletion of the murine Pten tumor suppressor gene leads to metastatic prostate cancer. Cancer Cell 4: 209–221. 14522255 10.1016/s1535-6108(03)00215-0

[WASSEFGAD269522C55] Watson PA, Ellwood-Yen K, King JC, Wongvipat J, Lebeau MM, Sawyers CL. 2005 Context-dependent hormone-refractory progression revealed through characterization of a novel murine prostate cancer cell line. Cancer Res 65: 11565–11571. 16357166 10.1158/0008-5472.CAN-05-3441

[WASSEFGAD269522C56] Wei Y, Xia W, Zhang Z, Liu J, Wang H, Adsay NV, Albarracin C, Yu D, Abbruzzese JL, Mills GB, 2008 Loss of trimethylation at lysine 27 of histone H3 is a predictor of poor outcome in breast, ovarian, and pancreatic cancers. Mol Carcinog 47: 701–706. 18176935 10.1002/mc.20413 PMC2580832

[WASSEFGAD269522C57] Wilson BG, Wang X, Shen X, McKenna ES, Lemieux ME, Cho YJ, Koellhoffer EC, Pomeroy SL, Orkin SH, Roberts CW. 2010 Epigenetic antagonism between polycomb and SWI/SNF complexes during oncogenic transformation. Cancer Cell 18: 316–328. 20951942 10.1016/j.ccr.2010.09.006 PMC2957473

[WASSEFGAD269522C58] Woodhouse S, Pugazhendhi D, Brien P, Pell JM. 2013 Ezh2 maintains a key phase of muscle satellite cell expansion but does not regulate terminal differentiation. J Cell Sci 126: 565–579. 23203812 10.1242/jcs.114843

[WASSEFGAD269522C59] Wu X, Wu J, Huang J, Powell WC, Zhang J, Matusik RJ, Sangiorgi FO, Maxson RE, Sucov HM, Roy-Burman P. 2001 Generation of a prostate epithelial cell-specific Cre transgenic mouse model for tissue-specific gene ablation. Mech Dev 101: 61–69. 11231059 10.1016/s0925-4773(00)00551-7

[WASSEFGAD269522C60] Xu K, Wu ZJ, Groner AC, He HH, Cai C, Lis RT, Wu X, Stack EC, Loda M, Liu T, 2012 EZH2 oncogenic activity in castration-resistant prostate cancer cells is Polycomb-independent. Science 338: 1465–1469. 23239736 10.1126/science.1227604 PMC3625962

[WASSEFGAD269522C61] You JS, Jones PA. 2012 Cancer genetics and epigenetics: two sides of the same coin? Cancer Cell 22: 9–20. 22789535 10.1016/j.ccr.2012.06.008 PMC3396881

