# Supplementary material for: Impaired PRC2 activity promotes transcriptional instability and favors breast tumorigenesis
Source: Genes Dev. 2015 Dec 15;29(24):2547–62. doi: 10.1101/gad.269522.115 (PMC4699384; doi:10.1101/gad.269522.115)
Supplement: Supplemental Material [file supp_29_24_2547__index.html]

Impaired PRC2 activity promotes transcriptional instability and favors breast tumorigenesis — Supplemental Material 

# Impaired PRC2 activity promotes transcriptional instability and favors breast tumorigenesis

## Supplemental Material

**Files in this Data Supplement:**

- Adobe PDF - SuppMaterial.pdf
