# Supplementary material for: Impaired PRC2 activity promotes transcriptional instability and favors breast tumorigenesis
Source: Genes Dev. 2015 Dec 15;29(24):2547–62. doi: 10.1101/gad.269522.115 (PMC4699384; doi:10.1101/gad.269522.115)
Supplement: Supplemental Material [file supp_gad.269522.115_SuppMaterial.pdf]

Supplemental Figure Legends

Supplementary Figure 1 (Related to Figure 1)

(A) anti-Ezh2, -Ezh1 and -Suz12 IHC of normal mouse prostate and Hi-Myc invasive prostate cancer. (B) Western blot showing loss of Ezh2 protein in *Hi-Myc*; *Ezh2* Δ/Δ prostate adenocarcinoma. (C) HE staining and IHC of different proteins as indicated on top in WT (normal prostate), Hi-Myc and Hi-Myc Ezh2-KO invasive adenocarcinoma. (C) IHC of histone H3, Ezh1 and smooth muscle actin (SMA) in WT (normal prostate), Hi-Myc and Hi-Myc Ezh2-mutant invasive adenocarcinomas. (D) Left panel: western blot of Ezh2 and Lamin B1 (loading control) on whole lysates from wild type prostate, *Pten*-mutant and double *Pten*/*Ezh2*-KO prostate cancers. Right panel: HE staining of a normal prostate, *Pten*-mutant and double *Pten*/*Ezh2*-KO prostate adenocarcinomas.

Supplementary Figure 2 (Related to Figure 2)

(A) Box plots of *EZH2* and *Ki67* mRNA abundance in primary versus metastatic prostate cancer (data from Taylor et al., 2010). p-value (Mann-Whitney) and mean log2-expression difference between metastatic and primary cancer are shown on each graph (B) Left panel: heat-map of hierarchical clustering of the most-significantly varying transcripts in breast cancers (BCa) of the Curie cohort. Samples are arranged horizontally and transcripts vertically. The cluster containing *EZH2* transcript is shown in greater detail on the right. Right panel: gene ontology (DAVID: http://david.abcc.ncifcrf.gov/) of the *EZH2* cluster showing the 20 most significantly enriched categories, their fold enrichment and corresponding p-value. (C) Top panel: graphical representation of copy-number variations along human chromosome 7 in a Curie cohort of breast cancers. The different samples are displayed horizontally and color-coded according to their ploidy and molecular subtypes (Basal, HER2-positive, Luminal A and Luminal B) as shown above. Copy-number events (losses, gains and amplifications) are displayed vertically following the color code as shown on the right. The positions of the centromere (black line) and of *EZH2* locus (red line) are highlighted. Bottom table represents numbers and corresponding percentages of losses, gains and amplifications of the *EZH2* locus in the different molecular subtypes. (D) Dot plots of Ezh2 signal intensity (arbitrary units) from the western blots shown in Figure 2B, as a function of the growth rate in sh-scr. (blue) and sh-Ezh2 (red) cells. (E) Correlation plot of H3K27me3 vs. EZH2 staining intensities across the same two patient-derived xenografts (PDX) shown in Figure 4. Intensity values were normalized to the control (untreated) condition. The corresponding coefficient of determination (R^2) and p-value are shown.

Supplementary Figure 3 (Related to Figure 3)

(A) ROC analysis of the prognostic association to death from breast cancer of *EZH2*, *ORC6* and the proliferation metagene. The doted line represents the diagonal. The corresponding area under de curve (AUC) and p-values are displayed. Data is from the METABRIC dataset. (B, C) Comparison of ROC-curves of *EZH2* (B) and *ORC6* (C) before (green) and after (purple) adjustment to the proliferation metagene. Negative association to outcome is displayed below the diagonal for better clarity. The corresponding AUCs and p-values are displayed. (D) Kaplan-Meier plot of overall survival for patients of the TCGA cohort having primary tumors with normal, hemizygous loss or gain of *EZH2*. Hazard ratio (HR) and p-value between highest and lowest survival group are displayed.

Supplementary Figure 4 (Related to Figure 4)

(A) Western blot showing near-complete loss of H3K27me3 mark in the parental MDA-MB-231 clone at various times of 1 μM UNC1999 treatment. (B) Proliferation curve of control (DMSO-treated) and UNC1999-treated cells after 8 days of UNC1999 treatment. (C) 3D invasion assay of control (DMSO-treated) or UNC1999-treated cells (see Figure 4 for experimental details). Images show representative phalloidin-labeled spheroids collected at T0 (inset) or T2 (scale bars: 200 µm). Data represent mean invasion area in type I collagen at T2 normalized to the mean invasion area at T0 ± SEM (*n*=2; 23 and 21 spheroids were analyzed for control and UNC1999-treated cells respectively). Red bars indicate mean ± SD. The p-value of the two-tailed unpaired t test is indicated.

Supplementary Figure 5 (Related to Figure 5)

(A) Heat map showing changes in the transcriptome following OHT addition in iMEFs at different time-points after treatment. Values for each gene are median-centered. (B) Correlation plot of normalized read counts for H3K27me3 between Ezh1-rescue *vs.* Ezh2-rescue conditions at common target peaks. The corresponding coefficient of determination (R^2) and p-value are shown.

Supplemental Table Legends

Supplementary Table 1 (related to Figure 2)

Normalized staining intensities of Ki67, H3K27me3 and EZH2 are shown for each treatment condition of the two analyzed tumorgrafts (HBCx34 TamR and HBCx22 TamR). Each value represents the mean from 3 independent experiments. 2 sections of each sample were analyzed giving a total of 6 measurements for each condition.

Supplementary Table 2 (related to Figure 3)

Summary of the mutations and copy number alterations in genes encoding core PRC2 components in 58 breast cancer metastases.

Supplementary Table 3

Primer sequences.

Supplementary Table 4

Primer sequences used for qPCR analysis on single cells.

Wassef_FigS1

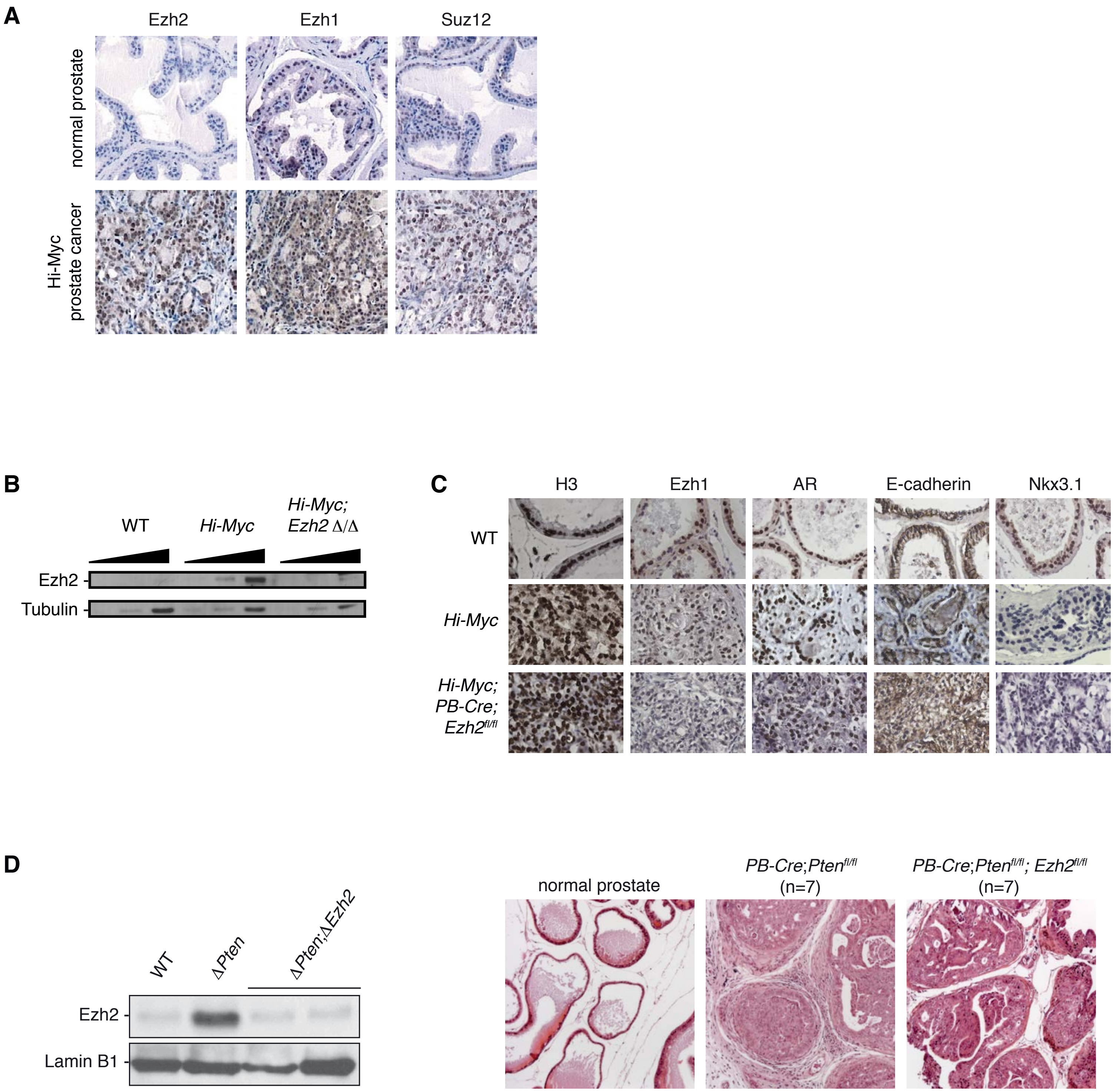

Wassef_FigS2

A

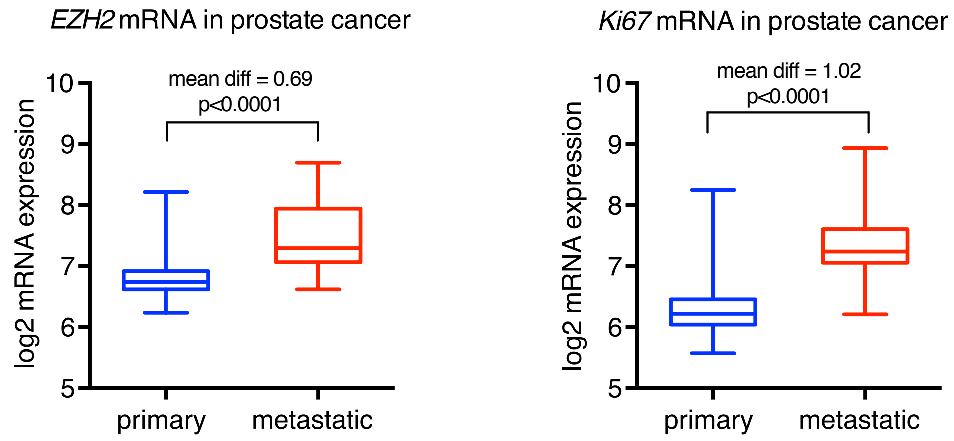

B

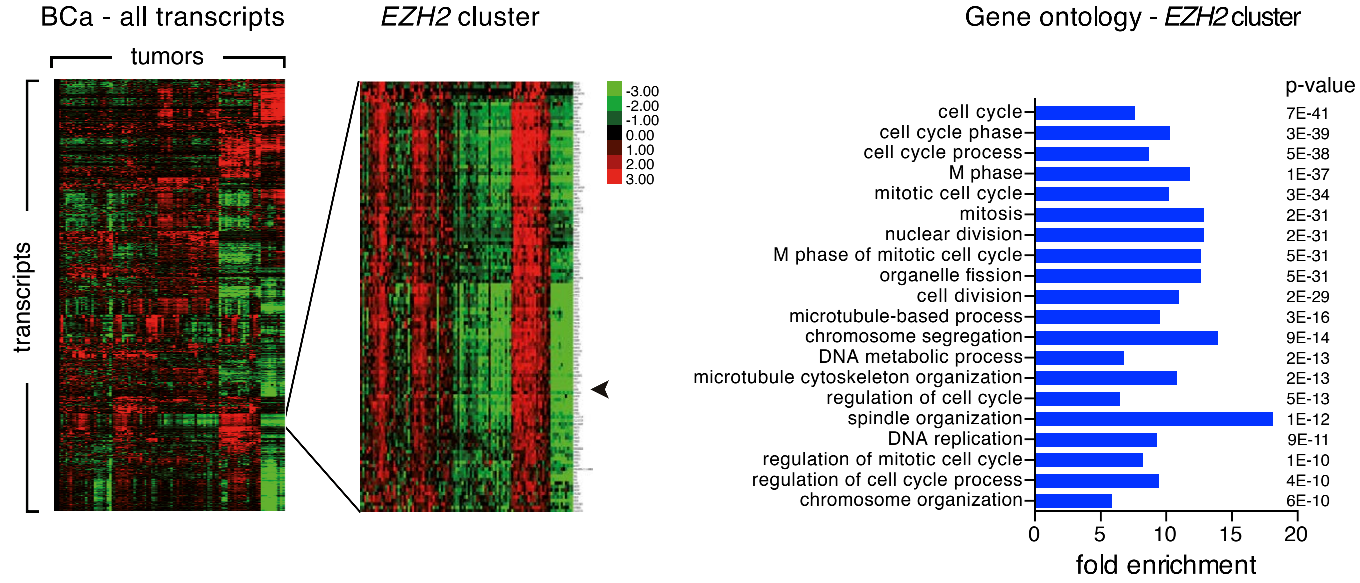

C

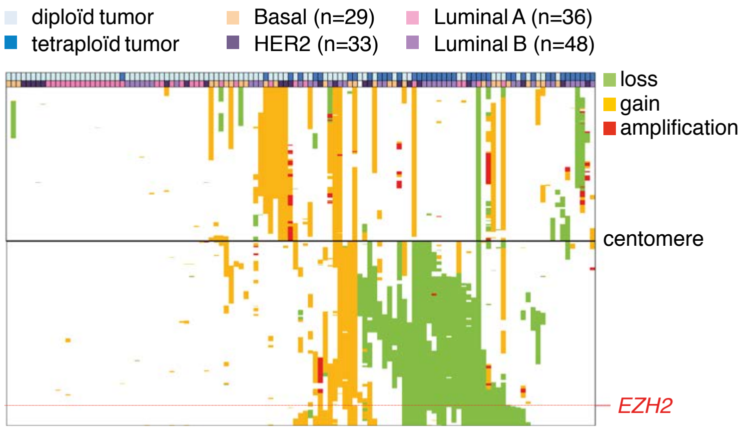

		status of EZH2 locus					
Subtype	nb of cases	loss	%	gain	%	amplification	%
Basal	29	4	14%	2	7%	0	0%
Her2	33	3	9%	4	12%	0	0%
Luminal A	36	5	14%	0	0%	0	0%
Luminal B	48	9	19%	3	6%	0	0%
TOTAL:	146	21	14%	9	6%	0	0%

diploid tumor: loss: ≤ 1 ; gain: ≥ 3 ; amplicon: ≥ 6
tetraploid tumor: loss: ≤ 2 ; gain: ≥ 6 ; amplicon: ≥ 8

D

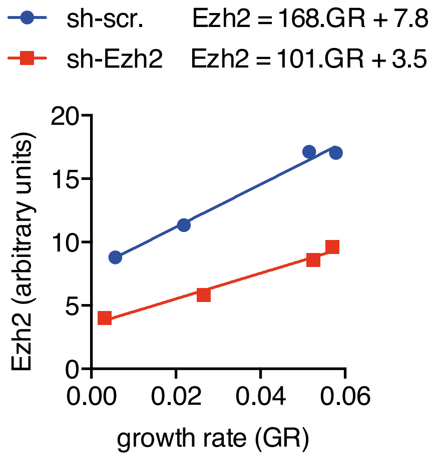

E

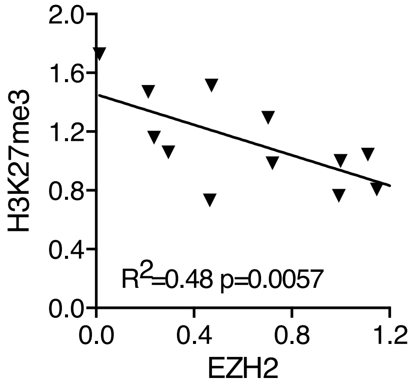

Wassef_FigS3

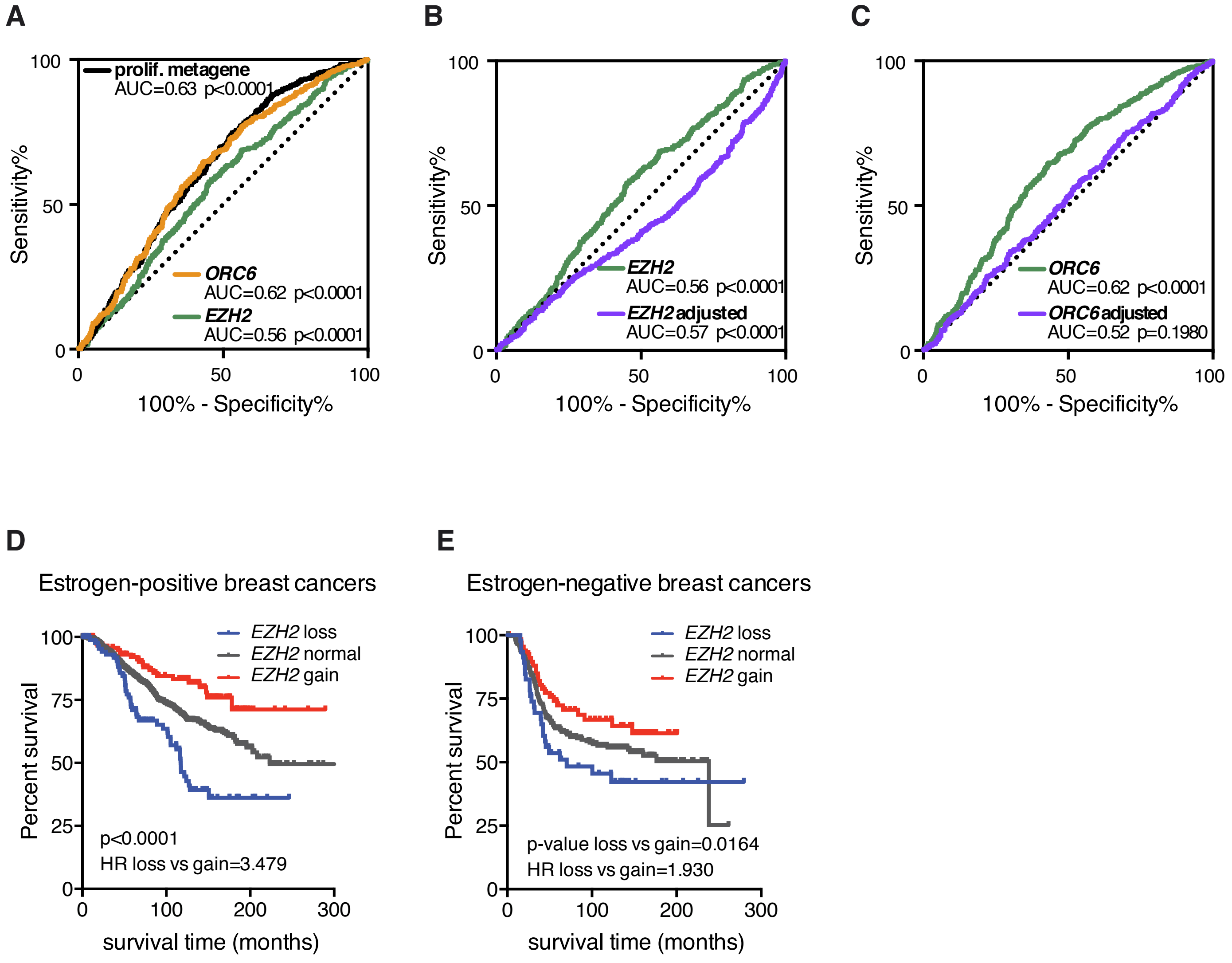

Wassef_FigS4

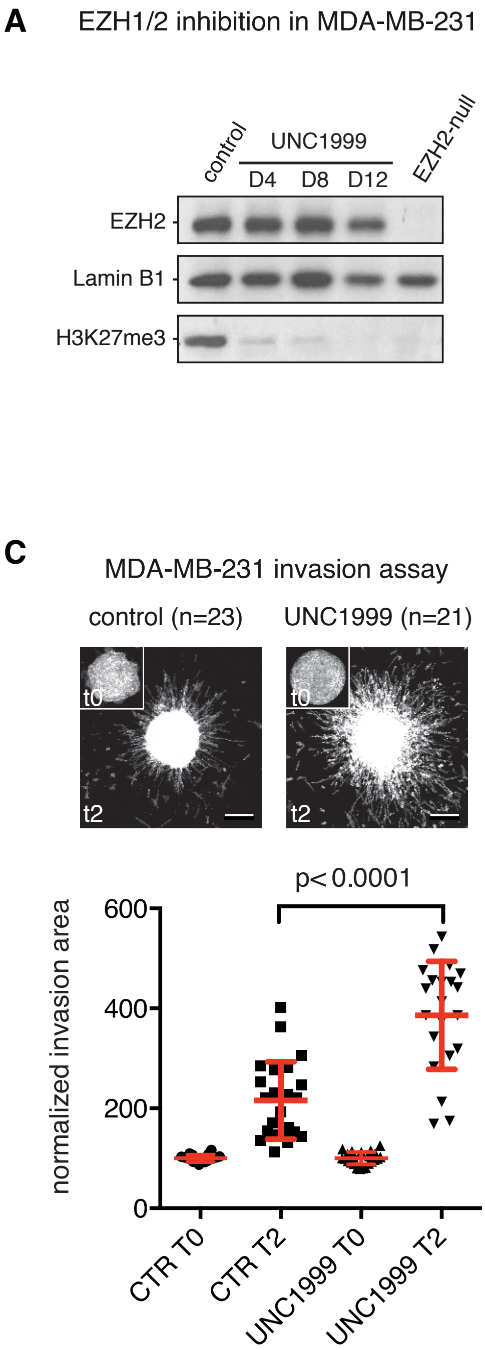

Wassef_FigS5

A

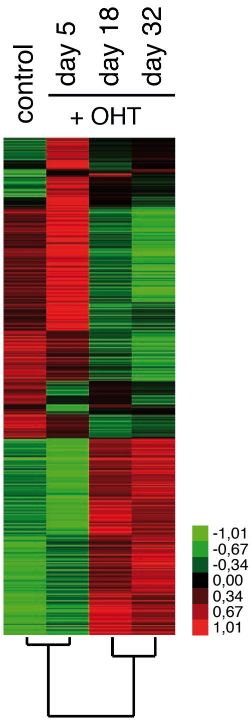

B

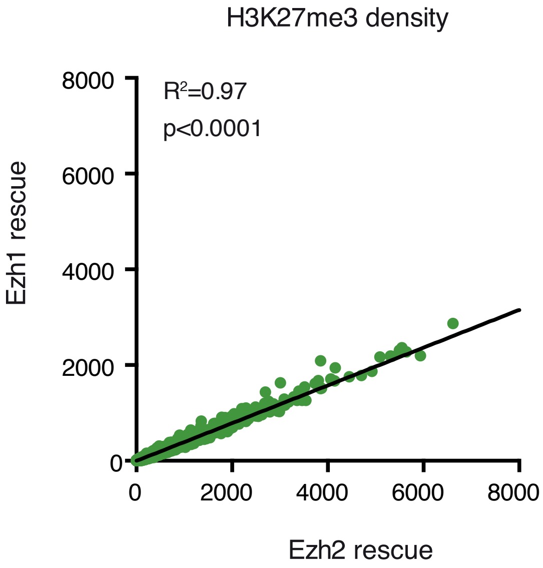

Table S1

treatment/tumorgraft	normalized signal values		
HBCx34 TamR	Ki67	H3K27me3	EZH2
Control	1.00	1.00	1.00
Everolimus	0.59	0.98	0.72
Femara	1.03	0.76	0.99
Tamoxifene	0.43	0.73	0.47
Evero+Tam	0.29	1.06	0.30
Fulvestran	0.20	1.16	0.24
Evero+Fulv	0.07	1.73	0.01
HBCx22 TamR	Ki67	H3K27me3	EZH2
Control	1.00	1.00	1.00
Everolimus	0.59	1.29	0.70
Femara	1.06	0.81	1.15
Tamoxifene	0.83	1.04	1.11
Evero+Tam	0.46	1.51	0.47
Fulvestran	0.80	0.78	1.31
Evero+Fulv	0.21	1.47	0.21

Table S2

Patient ID	Targeted NGS: EED, SUZ12, EZH2	EZH2 CNV	SUZ12 CNV	EED CNV
BCa-met-1		N	GAIN	HETLOSS
BCa-met-2		N	GAIN	HETLOSS
BCa-met-3		N	GAIN	N
BCa-met-4		GAIN	GAIN/a	HETLOSS
BCa-met-5		GAIN	HETLOSS	GAIN
BCa-met-6		N/HETLOSS	HETLOSS	GAIN
BCa-met-7		N	HETLOSS	GAIN/A
BCa-met-8		N	HETLOSS	HETLOSS
BCa-met-9		N	HETLOSS	HETLOSS
BCa-met-10		N	HETLOSS	HETLOSS
BCa-met-11		N	HETLOSS	HETLOSS
BCa-met-12		N	HETLOSS	HETLOSS
BCa-met-13		N/HETLOSS	HETLOSS	HETLOSS
BCa-met-14		N	HETLOSS	HETLOSS
BCa-met-15		GAIN	HETLOSS	HETLOSS
BCa-met-16		HETLOSS	HETLOSS	HETLOSS
BCa-met-17		HETLOSS	HETLOSS	N
BCa-met-18	EZH2 Ex18 c.2075C>T 35% (240/131) Ala692Val	N	HETLOSS	N
BCa-met-19		N	HETLOSS	N
BCa-met-20		N	HETLOSS	N
BCa-met-21		N	HETLOSS	N
BCa-met-22	SUZ12 Ex1 c.98C>T 53% Ala33Val	N/HETLOSS	HETLOSS	N
BCa-met-23		N	HETLOSS	N
BCa-met-24		GAIN	HETLOSS	HETLOSS
BCa-met-25		GAIN	HETLOSS	N
BCa-met-26		N	HETLOSS	N
BCa-met-27	SUZ12 Exon1 14% (7/47) c.61G>C p.Gly21Arg	HETLOSS	N	GAIN
BCa-met-28		N	N	GAIN
BCa-met-29		N/HETLOSS	N	GAIN
BCa-met-30	EZH2 -1 of exon 11 (splicing) 27% (428/161) chr7:148514484 G>C	HETLOSS	N	HETLOSS
BCa-met-31		N	N	HETLOSS
BCa-met-32		HETLOSS	N	HETLOSS
BCa-met-33		N	N	HETLOSS
BCa-met-34		N	N	HETLOSS
BCa-met-35		N	N	HETLOSS
BCa-met-36		N/HETLOSS	N	HETLOSS
BCa-met-37		HETLOSS	N	N
BCa-met-38		HETLOSS	N	N
BCa-met-39		N	N	N
BCa-met-40		GAIN	N	N
BCa-met-41		N	N	N
BCa-met-42		GAIN	N	N
BCa-met-43		GAIN	N	N
BCa-met-44		HETLOSS	N	N
BCa-met-45		N	N	N
BCa-met-46		N	N	N
BCa-met-47		N	N	N
BCa-met-48		N	N	N
BCa-met-49		N	N	N
BCa-met-50		N	N	N
BCa-met-51		N	N	N
BCa-met-52		N	N	N
BCa-met-53		N	N	N
BCa-met-54		N	N	N
BCa-met-55		N	N	N
BCa-met-56		N	N	N
BCa-met-57		N	N	N
BCa-met-58		N	N	N

Table S3

Name	Application	Sequence	Reference
**p16_fw**	RT-qPCR	GTGTGCATGACGTGCGGG	Bruggeman et al., 2005
**p16_rev**	RT-qPCR	GCAGTTCGAATCTGCACCGTAG	Bruggeman et al., 2005
**p19_fw**	RT-qPCR	GCCGCACCGGAATCCT	Bruggeman et al., 2005
**p19_rev**	RT-qPCR	TTGAGCAGAAGAGCTGCTACGT	Bruggeman et al., 2005
**p21_fw**	RT-qPCR	TTGCACTCTGGTGTCTGAGC	He et al., 2011
**p21_rev**	RT-qPCR	TGCGCTTGGAGTGATAGAAA	He et al., 2011
**TBP_fw**	RT-qPCR	ATCCCAAGCGATTTGCTG	this study
**TBP_rev**	RT-qPCR	CCTGTGCACACCATTTTTCC	this study
**Ezh2_fw**	RT-qPCR	AATACATGTGCAGCTTTCTGTTC	this study
**Ezh2_rev**	RT-qPCR	ACGAATTTTGTTGCCCTTTC	this study
**foxf1a_fw**	ChIP	CCCGCGGGCTTCTCTACTCTTATT	this study
**foxf1a_rev**	ChIP	GGCAGGAAGTTTACAAGGCTCAACG	this study
**p16-prom_fw**	ChIP	GATGGAGCCCGGACTACAGAAG	Bracken et al., 2007
**p16-prom_rev**	ChIP	CTGTTTCAACGCCCAGCTCTC	Bracken et al., 2007
**p19-prom_fw**	ChIP	AAAACCCTCTCTTGGAGTGGG	Bracken et al., 2007
**p19-prom_rev**	ChIP	GCAGGTTCTTGGTCACTGTGAG	Bracken et al., 2007
**p21-prom_fw**	ChIP	AGCAGCAAAATCGGAGCTCAGCAG	this study
**p21-prom_rev**	ChIP	TGAAGCAGCCCCACCTCTTCAA	this study
**actin_fw**	ChIP	CCCAACACACCTAGCAAATTAGAACCAC	this study
**actin_rev**	ChIP	CCTGGATTGAATGGACAGAGAGTCACT	this study

Table S4

Target	Forward primer	Reverse primer
1700019D03Rik	GCCAGGGGCATGAGAACA	GCAAGCACTAGTCCCTTTCACTA
1700028P14Rik	AACTCTCCGCTCACATCACA	TGGGGAAGGCTTCTCTGTTAC
2310005G13Rik	CAAGAGTCGGCCCATATCAC	TCCACCAGAGACCTATGGTAAC
2310042E22Rik	TCAGTCTCCCATCTGGTTCC	AAAGCCATGACAGGAAAGCA
4921506M07Rik	GGATAGTGCTCTTCCCACAAC	TGCATGAATCCTCTTGGTTCC
4930522H14Rik	GGCATCTGCTTTGTGGTTCC	GGTTTGCATGATCCTTGCTTCA
5830473C10Rik	CATGTCAGTGGGCAGGATCA	CCTGAAGAAACTGAGCCACCATA
9130409I23Rik	AATGATCGACGTCGGGAGATA	CAGGTTAGGGTCCGGTTTCA
A1cf	GGGGATTTGCCTTTGTGGAA	AGGATGTCCCCACAACTGAA
Abcb4	AAGGGCACCACTGAACTCAA	GAACATTCCAACCTTGTCACCAA
Acot5	GCGCCACGCTCTTTCTG	CAGAAGGCCACCTCCAACT
Acrbp	AGTTCCTCCCACTTCCATGAC	CTCAGGCCAAGGCTGGAA
Actb	CCCTAAGGCCAACCGTGAAA	AGCCTGGATGGCTACGTACA
Agr3	CACATGGGTGCAAACTTACGAA	AGTCCTCCAGGTGGTGAATAAC
Art2a-ps	CTACTCAAGCAGCCAACGTTA	AAGACCGAGGAGAACCACAA
Bmp10	TCCATGCCGTCTGCTAACA	CCGGAGCCCATTAAAAGTGAC
Bpi	AGAGACTCTGTGTCCTCCAAAC	AACCGAGGTCACATCATCCA
Bub1	AGCCATCAAGACCAAGACAGAA	AGCAAAGGCTCCTTCTCCAA
Calr3	CAAGCCGAGTGACTGGAACA	GTCGATGCCCTCAGCTTTCA
Ccdc146	TTAGCTGAAACACCAGCTTTCC	ACTCTGCCATTCTGGTTCCA
Cckar	CTGGATCAACCTCAGCCTTCA	CCCAGCACACTGAGAAGGAA
Ccnb1	GCCTGAGCCTGAACCTGAA	TCGGGCTTGGAGAGGGATTA
Ccne1	CTGTGAAAAGCGAGGATAGCA	TGTTAGGGGTGGGGATGAAA
Ccnf	TGTGGCCTTCTCCACAGAA	CCAACTTCACAGCAGCTTCA
Cd200r4	ATGACAGTTCATCTTCTCTGACA	AGGCAGGTGTCTGTGTTTTA
Cd72	GCGGCTTAGAGGAGTTGCTA	ACAATGTCGGCTTTGAGAGTCA
Cdh22	CATCGTGGTTACTGACGTCAAC	GGCTGACTCCTGTATGCTGAA
Cdkn3	CAGACGAAGAACCTGTTGATGAA	ATTCACTCGCGACAGAGGTA
Cmtm2a	TCCCTTCGTCTTCTGGCCTA	ATATGCCACCTCCAAGGAACAC
Cnga1	TACAAAAGGCGAGGACCATCA	CTTTGTTGCTGCTGTTGTTGAC
Cpa6	ATCGGTCCTGCCTTTTGTCA	CATGGCTGGGTCAGTCTTGTA
Cpb2	GGCACAGACCTGAACAGGAA	TTCAGAGCAGGAGGAACTTGAC
Ctxn2	TGAAGATCCGAGGTTTTGGAA	TGGTGGCTCTTCAGAGTTCC
Defb26	GGTTGCTAACTCAGGGGAAC	TGACATCGGACTCTGCAGTA
Dgat2l6	GCCCTTGTCTGTGCTCTTCTTA	TACCCAAGCTGAACGCCTAC
Dntt	CAGAGGGGATTCCTTGCCTA	GCTTCAGAACTTTCTCCATCTTCA
E2F1	AGCTCATTGCCAAGAAGTCC	TTCAAGCCGCTTACCAATCC
Fam159b	CAGCTACTTCCCCTACAAGCA	AGCGATTCCCAATCCAACCA
Fam71f1	CCGTGGTGTTCGAAAGCAA	GCCATTGTGTTGGAAGCCTTA
Fras1	GAGCCACAGCTGATCAACATAC	TGCTGACGTGGAGAACTTCA
Gapdh	AGACGGCCGCATCTTCTT	TTCACACCGACCTTCACCAT
Glyat	CAAGGTGCACAGATGCTACA	TCATGTGATAGACGGTCCCATA
Gm10857	CTCCCATGTGCTAGGTCTCA	ATCTCTGAGAGCCGTCAACA

Gm14085	GGAAGTGACACAGGGACTCA	GCCTTCGTGAAAGGCTGAAA
Gm4776	TTCCTGTGCAAAGCTCAGAC	CAGCACTCCAGCAATGCATA
Gpr50	AGACCTGATCGGGAACTCCA	CAGAGAGGCTGGCCACAAA
Hao2	GCTGGATTTGGAGGCAAACA	TAGACAGGGGAGTAGGAAGTCC
Homer2	AGCTCATGTCCGAGTGTGAA	GACCGCACTTTGTCTTCCAA
Hoxb9	TGCGAAGGAAGCGAGGAC	TCTAGCTCCAGCGTCTGGTA
Hprt	CAGTACAGCCCCAAAATGGTTA	AGTCTGGCCTGTATCCAACA
Hpse2	TCAGCTGGAGGCACAAACAA	CCCTGATTGGCCAACATTCCTA
Igf2bp3	TTGAGCACTCGGTCCCTAAA	ATTGTAAGTGGGGCGGGATA
Il10	AAAGGACCAGCTGGACAACA	TAAGGCTTGGCAACCCAAGTA
Il7	TTGTTCTGCTGCCTGTCAC	TTCGGGCAATTACTATCAGTTCC
Irs4	GGACTTTGCCAGACGAGACTA	GTTTGGTTTTGGTGGCAGTGTA
Lgi1	CTGAAATCTCAGAAGGGAGTTTCTTA	CACATCAAAGGAGTTCGACGTA
Mctp2	CGAGGATCCCAGTGAAAGACA	ATCCCCACATCCTTCACATCC
Mdh1b	GGCTTGCTTTTGGGAGGATAC	ACTCCGTCGTCATGTTGGAA
Mep1a	TCTTAAAGGCGACAGCCTCA	TGGAACTTCAGTCCGGTTCA
Ezh2	GGGCAACAAAATTCGTTTTGCTA	TCCTGTGGTCACCATTAACCA
Nipal2	CACATTGTGGTCCTGCTCAC	ATGCCTGAGACAGCCTTCA
Npy1r	TTCGGCCCACTCTGCTTTATA	GTCCCGGATCTTGTCCATCA
Olfr556	TGTTCTGGCTGCTGTGGATA	GGAGCTGTCTCCTGAACTGAA
Onecut1	ACCTTCCGGAGGATGTGGAA	TCCCGTGTTCTTGCTCTTTCC
Pip5k1b	ATCCACATGACAGGACTACCC	GTCCCCTCTTCCAAGGTGAA
Pou1f1	GAAGGTGGGAGCAAACGAAA	AGTGTCTCTCCAGAGCATCC
Prkcq	GGCGACTTAATGTACCACATCC	AGGATGACCTCAGCAGCATAA
Prss38	GTGCTGCAGACATCAAGACA	ACCAGGTCTGGTTCTGCTTA
Psg16	AAGCACACGTGCAAATCCAA	TGAAGACCACTGAGCTCTGTAC
Rad21	GCACTCAGCAGATGCTTCA	TCGACACAGCTCAAGCAAAC
Rad51	AACCCATTGGAGGGAACATCA	GATTCTGGTCTCCCCTCTTCC
Rad9b	AAGTGATGCACGCACCTCTA	CAAGAGCATGTCTTCAACACTCAA
Rdh12	TTGAGACCCACTTTGGAGTCA	GACTCCTTCAGCCTCTCCAA
Rdh18-ps	TTGGTGACTGACTGCATGGAA	GAACTTGGCATCCCAGCCTA
Rgag4	AAATGCCCTTCGAGGAGAGAA	TTGCCCCGCAAGGAATTAAC
Rgs22	CCCCAGCTGGAGAGAAAATACA	AATCCTCCCAAGGCTCCAAC
Rnf128	ATTTGGTGCGCATCCTAACC	GGGGCAAGTCCTGTGTTCTA
Rorb	GGAGCTGTGTCAGAACGATCA	GAAGGCACGACACATTCTCAC
Rrm2	AGCAAAGCTGCGAGGAGAA	CATCCTCAACGCTGGGGTTA
Rxfp1	TCAGTCGAATTTCCCCACTCA	ACGGGTGAGGGCATTATTCA
Scgn	ATTCTGGCGCTCCAGGAAAA	CTCAAAGTCCCTCTTCCTTTCTTCA
Slc26a3	TGACATCGTCTCTGGCATCA	GATGTTGACCAGCAGAGCAAA
Slc2a10	CTTCAACTGGGCAGCTAACC	CCATAGAGCAGGAAGGTCCAA
Slc7a12	ACTTGGCAGTTTTGACATCCC	TGTTCATCCATGTGACACCAAC
Sult1c1	CCTATGACCGACACCCTTTCA	GGCATTTTGTTAGCCAGATCCA
Svs6	CAATGCTCCTTGTTCTGGTGAC	TCTTCAATTGCTTGCGAGAACC
Taar4	ACTTCAAGCAGCTCCACTCC	ACCACGCAACTCAACAGGAA
Tac2	GACCAAAGGAGACATCACTTCCA	TGTTCCTCTTGCCCATAAGTCC
Tdh	GGGTATCCAAGGTCCACACA	CGCAGGCACCGAAAATCTAA

Tdo2	GGTGTAAACAGAGCCAGCAA	TCTGAAGTTCTTGCGCATTCA
Top2a	GCCAAGAGCTTTGGATCAAC	GCTTTCCACAATACCACAACC
Tspan7	GCCTCTTTGGATGCTTTGCTAC	GAACACCAGGGACAGGAACA
Upk1b	AAGAACAACGGGGTCACCAA	CAGTCTGACGGACCGTTTACA
Wnt7b	CGCCTCATGAACCTTCACAA	CTGACACACCGTGACACTTAC
Wnt8a	CCTGGTGAACCTTCACAACAA	GCTTCCTGAGATGCCATGAC
